# Microbiota-derived 3-IAA influences chemotherapy efficacy in pancreatic cancer

**DOI:** 10.1038/s41586-023-05728-y

**Published:** 2023-02-22

**Authors:** Joseph Tintelnot, Yang Xu, Till R. Lesker, Martin Schönlein, Leonie Konczalla, Anastasios D. Giannou, Penelope Pelczar, Dominik Kylies, Victor G. Puelles, Agata A. Bielecka, Manuela Peschka, Filippo Cortesi, Kristoffer Riecken, Maximilian Jung, Lena Amend, Tobias S. Bröring, Marija Trajkovic-Arsic, Jens T. Siveke, Thomas Renné, Danmei Zhang, Stefan Boeck, Till Strowig, Faik G. Uzunoglu, Cenap Güngör, Alexander Stein, Jakob R. Izbicki, Carsten Bokemeyer, Marianne Sinn, Alec C. Kimmelman, Samuel Huber, Nicola Gagliani

**Affiliations:** 1 II. Department of Medicine, University Medical Center Hamburg-Eppendorf, Hamburg, Germany; 2 Mildred Scheel Cancer Career Center HaTriCS4, University Medical Center Hamburg-Eppendorf, Hamburg, Germany; 3 Department of General, Visceral and Thoracic Surgery, University Medical Center Hamburg-Eppendorf, Hamburg, Germany; 4 Research Group Microbial Immune Regulation, Helmholtz Centre for Infection Research, Braunschweig, Germany; 5 I. Department of Medicine, University Medical Center Hamburg- Eppendorf, Hamburg, Germany; 6 Hamburg Center for Translational Immunology (HCTI), Hamburg, Germany; 7 III. Department of Medicine, University Medical Center Hamburg-Eppendorf, Hamburg, Germany; 8 Department of Clinical Medicine, Aarhus University, Aarhus, Denmark; 9 Institute of Clinical Chemistry and Laboratory Medicine, University Medical Center Hamburg-Eppendorf, Hamburg, Germany; 10 Newborn Screening and Metabolic Laboratory, Department of Pediatrics, University Medical Center Hamburg-Eppendorf, Hamburg, Germany; 11 Research Department Cell and Gene Therapy, Department of Stem Cell Transplantation, University Medical Center Hamburg-Eppendorf, Hamburg, Germany; 12 Bridge Institute of Experimental Tumor Therapy, West German Cancer Center, University Hospital Essen, University Duisburg-Essen, Essen, Germany; 13 Division of Solid Tumor Translational Oncology, German Cancer Consortium (DKTK Partner Site Essen) and German Cancer Research Center (DKFZ), Heidelberg, Germany; 14 Irish Centre for Vascular Biology, School of Pharmacy and Biomolecular Sciences, Royal College of Surgeons in Ireland, Dublin, Ireland; 15 Center for Thrombosis and Hemostasis (CTH), Johannes Gutenberg University Medical Center, Mainz, Germany; 16 Department of Internal Medicine III, Ludwig-Maximilians-University (LMU) Hospital, Munich, Germany; 17 Hannover Medical School (MHH), Hannover, Germany; 18 Hematology–Oncology Practice Hamburg (HOPE), University Cancer Center Hamburg, Hamburg, Germany; 19 Department of Radiation Oncology, Perlmutter Cancer Center, New York University Grossman School of Medicine, New York, NY USA

**Keywords:** Cancer therapeutic resistance, Tumour heterogeneity, Predictive markers, Chemotherapy

## Abstract

Pancreatic ductal adenocarcinoma (PDAC) is expected to be the second most deadly cancer by 2040, owing to the high incidence of metastatic disease and limited responses to treatment^1,2^. Less than half of all patients respond to the primary treatment for PDAC, chemotherapy^3,4^, and genetic alterations alone cannot explain this^5^. Diet is an environmental factor that can influence the response to therapies, but its role in PDAC is unclear. Here, using shotgun metagenomic sequencing and metabolomic screening, we show that the microbiota-derived tryptophan metabolite indole-3-acetic acid (3-IAA) is enriched in patients who respond to treatment. Faecal microbiota transplantation, short-term dietary manipulation of tryptophan and oral 3-IAA administration increase the efficacy of chemotherapy in humanized gnotobiotic mouse models of PDAC. Using a combination of loss- and gain-of-function experiments, we show that the efficacy of 3-IAA and chemotherapy is licensed by neutrophil-derived myeloperoxidase. Myeloperoxidase oxidizes 3-IAA, which in combination with chemotherapy induces a downregulation of the reactive oxygen species (ROS)-degrading enzymes glutathione peroxidase 3 and glutathione peroxidase 7. All of this results in the accumulation of ROS and the downregulation of autophagy in cancer cells, which compromises their metabolic fitness and, ultimately, their proliferation. In humans, we observed a significant correlation between the levels of 3-IAA and the efficacy of therapy in two independent PDAC cohorts. In summary, we identify a microbiota-derived metabolite that has clinical implications in the treatment of PDAC, and provide a motivation for considering nutritional interventions during the treatment of patients with cancer.

## Abstract (web summary)

Indole-3-acetic acid (3-IAA), a tryptophan metabolite derived from the gut microbiota, is associated with a better response to chemotherapy in pancreatic ductal adenocarcinoma (PDAC), and dietary interventions could have a role in the treatment of PDAC.

## Main

Polychemotherapy, either with 5-fluorouracil (5-FU), irinotecan and oxaliplatin in combination with folinic acid (FOLFIRINOX), or with gemcitabine and nab-paclitaxel (GnP), is considered the standard of care for patients suffering from metastatic PDAC (mPDAC)^3,4^. However, less than half of all patients are responsive to the therapy, and patients who do not respond (NR patients) suffer from pain and eventually die within weeks^3^. Genetic alterations in PDAC poorly explain the differences between patients who respond to therapy (responder (R) patients) and NR patients^5,6^, which leaves environmental factors—including the intestinal microbiota—as the potential mediators of chemotherapy efficacy. There is, therefore, an urgent need to identify environmental factors that might explain the differences between R and NR patients so that new concepts can be developed for future therapies.

The intestinal microbiota has been shown to induce a response to immunotherapy in patients with melanoma, and can be modulated by dietary habits^7–10^. In rare patients with localized PDAC who are long-term survivors, bacteria can translocate from the intestine into the tumour and control anti-tumour immune activation^11,12^. However, most patients suffering from aggressive immunotherapy-resistant mPDAC are treated with polychemotherapy, and it is at present unclear whether and how the microbiota or dietary habits affect its efficacy^1,13^.

## 3-IAA induces a response to chemotherapy

**Fig. 1:**
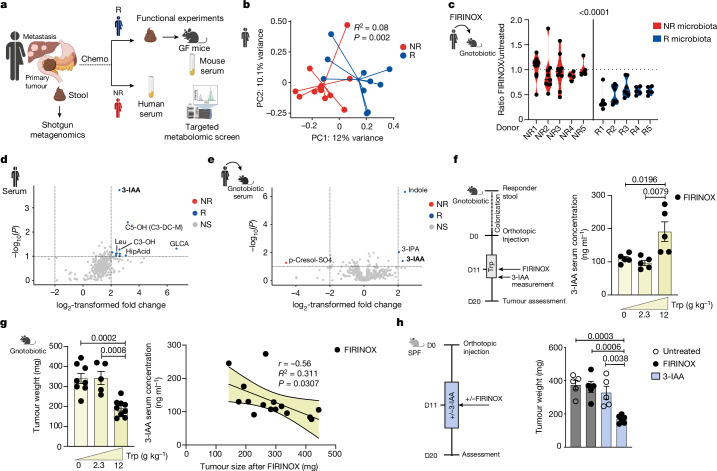
3-IAA induces a response to FIRINOX in mouse models of PDAC. **a**, The intestinal microbiota of 23 patients with mPDAC was sequenced before the start of chemotherapy (chemo). GF, germ-free. **b**, Principal coordinate analysis (PCoA), with Bray–Curtis dissimilarity matrix, of pre-treatment NR (*n* = 12) and R (*n* = 10; one patient was excluded after the quality control) microbiota. **c**, Gnotobiotic mice were colonized with five different R or NR microbiota, KPC cancer cells were orthotopically injected and mice were treated with FIRINOX or left untreated. Tumour weight is shown relative to the mean tumour weight of the untreated group of each experiment at day 20 after tumour cell injection (*n* = 9 NR1–3; *n* = 8 R2; *n* = 7 R1 and R3; *n* = 5 NR5, R4 and R5; *n* = 4 NR4; pooled from nine independent experiments). **d**,**e**, Volcano plots showing differentially abundant metabolites in the serum of three R and NR patients (**d**) and in the serum of gnotobiotic mice colonized with R or NR microbiota (*n* = 3 each, biological replicates) (**e**). NS, non-specified. **f**, R-microbiota-colonized gnotobiotic mice were fed with the indicated concentration of tryptophan, and the 3-IAA serum concentration at the fourth day of dietary intervention is shown (*n* = 5 each). **g**, Left, tumour weight of orthotopic KPC tumours (*n* = 8, 5 or 9) after FIRINOX treatment. Right, correlation between 3-IAA serum concentration and tumour weight of five randomly selected mice per dietary group from **f** (*n* = 15). **h**, SPF mice were orthotopically injected with KPC cells and treated with or without (+/−) 3-IAA and/or with or without FIRINOX, and tumour weight was assessed at day 20 of the experiment (*n* = 5 or 6). Each symbol represents one mouse. One out of two (**f**,**g**) or three (**h**) independent experiments is shown. Error bars indicate s.e.m. Significant *P* values are indicated and were determined by MANOVA (**b**), two-tailed nested *t*-test (**c**), fold change analysis and two-tailed *t*-test (**d**,**e**), simple linear regression and Pearson’s *r* (**g**) and one-way ANOVA with Tukey’s post-hoc test (**f**–**h**). Source data

**Extended Data Fig. 1:**
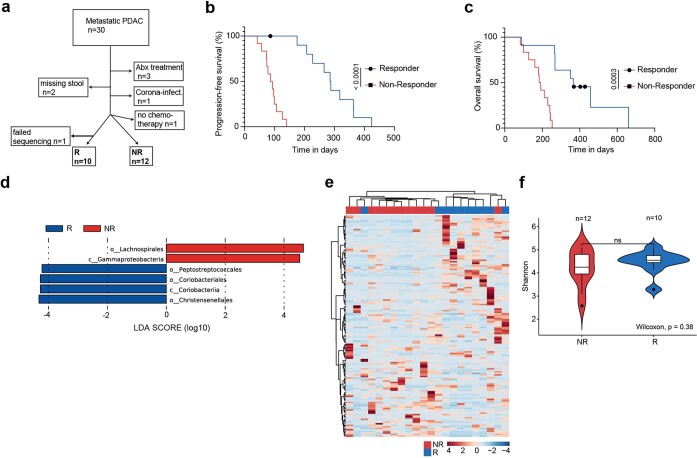
Survival and microbiota analysis of R and NR patients with mPDAC from the Hamburg cohort. **a**, 30 patients were recruited to the study. **b**,**c**, After exclusion of non-eligible patients as indicated, 23 patients could be analysed for PFS (**b**) and overall survival (OS) (**c**). PFS and OS are presented separately for responder (R, *n* = 11, blue) and non-responder (NR, *n* = 12, red) patients. **d**–**f**, After exclusion of one sample due to sample collection errors that led to sequencing failure, the microbiota of 22 patients was analysed. **d**, Microbiota LEfSe analysis showing linear discriminant analysis (LDA) score of bacterial taxa enriched in R or NR patients, respectively. **e**, Genus heat map with Ward clustering of patients. **f**, Microbiota diversity compared using Shannon index. Boxes indicate 25 to 75% of values (**f**) and error bars indicate median, significant p-values are indicated and were determined by Gehan–Breslow–Wilcoxon test (**b**,**c**) or two-tailed Wilcoxon test (**f**). Source data

**Extended Data Fig. 2:**
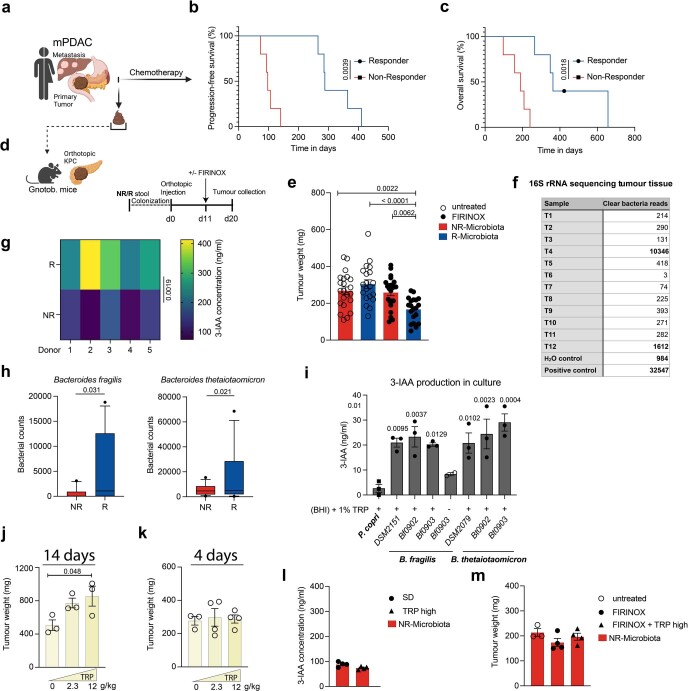
R microbiota induces a response to FIRINOX treatment. **a**, Microbiota of five R and NR patients with mPDAC was collected and transferred to gnotobiotic mice. Nine patients were treated with FIRINOX and one with GnP. PFS (**b**) and OS (**c**) of patients used for microbiota transfer experiments is depicted in Kaplan-Meier estimators. In four individual experiments with varying donors, one group of mice was colonized with R microbiota and one group was colonized with NR microbiota. After stool transfer, gnotobiotic mice (**d**) received orthotopic injections of KPC tumour cells and were either left untreated (n = 22 (NR) or 20 (R)) or treated once with FIRINOX (n = 23 (NR) or 20 (R)) at day eleven of the experiment. **e**, Tumour weight of orthotopic tumours is depicted for the respective experimental group at day 20 of the experiment. **f**, Randomly selected tumours from varying experiments of e were analysed for intratumoral bacteria using 16S rRNA sequencing (n = 12). Table shows indicated clean reads for different tumours (T1–12), H_2_O or positive control. **g**, 3-IAA was analysed in the serum of n = 3 to 5 randomly selected mice per experiment with different donors each. **h**, Metagenomic sequencing data was analysed for the abundance of a panel of 3-IAA-producing bacteria. Increased abundance of *B. fragilis* and *B. thetaiotaomicron* was detected in stool samples from NR or R patients during and before chemotherapy treatment, which were used for experiments in gnotobiotic mice (5 donors per group and 1–3 samples per patient, n = 12 per group). **i**, 3-IAA was quantified in supernatants of bacteria lacking (*Prevotella copri*) and having the ability (*B. thetaiotaomicron* and *B. fragilis*) to produce 3-IAA based on genomic predictions. The characterized strains included *P. copri* (DSM18205), *B. fragilis* (BF DSM2151, Bf0903, Bf0902) and *B. thetaiotaomicron* (DSM, Bt0903, Bt0902). Strains were all cultured in BHI containing 1% tryptophan, the strain Bf0903 was also cultured in BHI alone (n = 3 biological replicates). **j**, SPF mice were orthotopically injected with KPC cells. Mice were either supplemented with tryptophan-free, standard diet (2.3 g/kg TRP) or tryptophan-high diet (12 g/kg TRP) starting 7 days after tumour cell injection for a total of 14 days (n = 3). **k**, As in **j**, except dietary intervention was applied for a total of 4 days before standard diet was reintroduced. Tumour weight was assessed 9 days after dietary intervention (n = 3 or 4). **l**, NR microbiota-colonized mice (red) were fed either standard diet (SD) or tryptophan-high diet for 4 days (TRP high). Blood was drawn at day 4 of intervention and 3-IAA was measured in the serum using 3-IAA CLIA (n = 4). **m**, Tumour sizes of mice from **l** at day nine after treatment with FIRINOX are shown (n = 3 or 4). Each symbol represents one mouse, human or *in vitro* replicate. One experiment was performed (**f**,**i**–**m**) or four (**e**,**g**) independent experiments were pooled. Error bars indicate SEM, whiskers indicate 10% and 90% (**h**), significant p-values are indicated and were determined by Gehan-Breslow-Wilcoxon test (**b**,**c**), one-way ANOVA followed by Tukey’s (**e**,**m**) or Dunnett’s (**i**–**k**) post-hoc test, two-way ANOVA (**g**), two-tailed Wilcoxon paired-ranks test (**h**) or two-tailed t-test (**l**). Source data

We recruited 30 patients with mPDAC, of whom 23 did not receive antibiotics and provided enough sample material to allow the intestinal microbiota to be analysed before the beginning of chemotherapy treatment (Fig. 1a and Extended Data Fig. 1a). The cohort of patients was separated into R and NR patients mainly on the basis of radiological response or, in cases in which the disease stabilized or computed tomography (CT) scans were missing, on the basis of progression-free survival (PFS) and a decrease of serum tumour markers (see Methods for detailed criteria). R patients (*n* = 11) had a mean PFS of 40.9 weeks, which was significantly higher than the PFS of 12.8 weeks for NR patients (*n* = 12), and the overall survival was 51.9 weeks for R and 26.4 weeks for NR patients (Extended Data Fig. 1b,c). The microbiota of R patients was distinct from the microbiota of NR patients (Fig. 1b and Extended Data Fig. 1d–f). To study a potential cause–effect relationship between the microbiota and the response to chemotherapy, we transferred microbiota from the first ten recruited R and NR patients into gnotobiotic mice, followed by orthotopic injection of *Pdx1*-Cre, LSL-*KRAS*^G12D^, LSL-*Trp53*^R172H/+^ (KPC) pancreatic cancer cells (Extended Data Fig. 2a–d). Notably, four patients were treated with FOLFIRINOX and one patient was treated with GnP in both groups (R and NR). Irrespective of the original donor treatment, we observed smaller tumours in mice that were colonized with the microbiota of R patients, but not in mice colonized with the microbiota of NR patients, after chemotherapy treatment (5-FU, irinotecan and oxaliplatin; FIRINOX) (Fig. 1c and Extended Data Fig. 2e). Given the potential translocation of intestinal bacteria into PDAC tumours^11^, we next analysed the intratumoural bacteria by 16S rRNA sequencing. We could only detect intratumoural bacteria in 2 out of 12 tumours (Extended Data Fig. 2f), and thus hypothesized that the response to chemotherapy is indirectly controlled through circulating microbiota-derived metabolites.

To address this, we analysed serum from R and NR patients and matching colonized gnotobiotic mice and performed a targeted metabolomic screen using liquid chromatography coupled to mass spectrometry. We found that the tryptophan metabolite 3-IAA was the most significantly enriched metabolite in R compared to NR patients (Fig. 1d). In line with this, 3-IAA was also enriched in the serum of gnotobiotic mice that were colonized with R compared to NR microbiota (Fig. 1e and Extended Data Fig. 2g).

To characterize which gut bacteria in R patients could contribute to increased production of 3-IAA, we analysed the abundance of common 3-IAA-producing bacterial strains^14^ in the microbiota of R compared to NR patients. Out of the fifteen analysed 3-IAA producers, we found that *Bacteroides fragilis* and *Bacteroides thetaiotaomicron* were increased in R patients and confirmed their capacity to produce 3-IAA in vitro (Extended Data Fig. 2h,i).

We next wondered whether we could alter the serum levels of 3-IAA in mice and thereby affect the efficacy of chemotherapy by modulating the dietary concentration of tryptophan, the precursor of 3-IAA (ref. ^15^). Because tryptophan can impair the development of anti-tumour immune responses and thus promote tumour growth^16,17^, we first tested different lengths of dietary intervention. Fourteen days of tryptophan exposure promoted tumour growth, whereas a treatment period of only four days was not sufficient (Extended Data Fig. 2j,k). We therefore decided to choose a four-to-five-day intervention for the rest of our experiments to avoid the pro-tumorigenic effect^16,17^.

**Extended Data Fig. 3:**
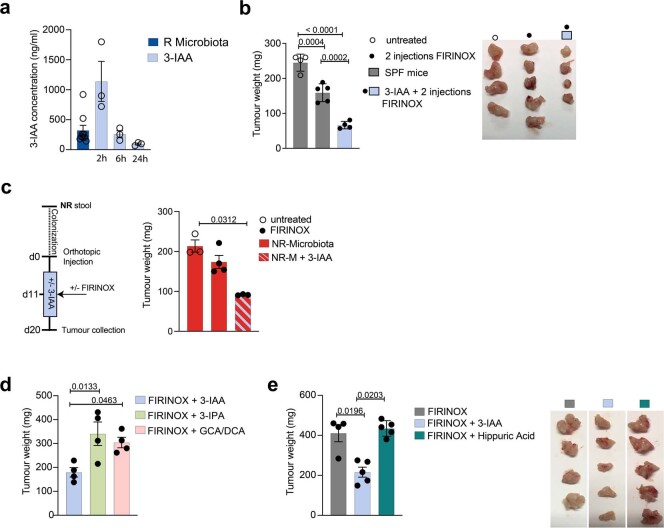
3-IAA induces a response to FIRINOX in mouse models of PDAC. **a**, 3-IAA serum concentration of SPF mice gavaged with 500 mg/kg 3-IAA was measured using CLIA at 2, 6 and 24 h after 3-IAA application (n = 3 mice). Unrelated serum concentration of R-microbiota-colonized gnotobiotic mice is shown as a control. **b**, SPF mice were orthotopically injected with KPC cells and received two treatments of FIRINOX or FIRINOX + 3-IAA (n = 5). Tumour weight at day 20 of the experiment is depicted in the statistic. **c**, NR-colonized gnotobiotic mice were injected with KPC tumour cells orthotopically. 9 days later, mice were substituted +/− 3-IAA for five days (d9–13) and treated +/− FIRINOX as depicted in the experimental scheme (n = 3 or 4). Tumour weight is shown at day 20 of the experiment. **d**, SPF mice were orthotopically injected with KPC tumour cells. At day 9 after tumour cell injection, mice were treated with either 500 mg/kg 3-IAA, 3-IPA or 250 mg/kg GCA and 250 mg/kg DCA for five consecutive days (n = 4). FIRINOX treatment was applied at day 11. Tumour weight is shown at day 20 of the experiment. **e**, As in **d**, except mice were treated with FIRINOX, FIRINOX + 3-IAA (500 mg/kg) or FIRINOX + hippuric acid (500 mg/kg) (n = 4 or 5). Each symbol represents one mouse. One experiment (**e**) or one out of two independent experiments is shown (**a**–**d**). Error bars indicate SEM, significant p-values are indicated and were determined by Kruskal–Wallis test followed by Dunn’s post-hoc test (**a**,**c**,**e**), or one-way ANOVA followed by Tukey’s (**b**) or Dunnett’s (**d**) post-hoc test. Source data

Second, we observed that four days of a high-tryptophan diet was already sufficient to increase the concentration of 3-IAA in the serum of gnotobiotic mice that were colonized with R microbiota (Fig. 1f). Moreover, when this dietary intervention was combined with FIRINOX, we observed a decreased tumour weight (Fig. 1g). In addition, 3-IAA serum levels correlated inversely with tumour weight (Fig. 1g). Notably, these tryptophan-mediated effects—that is, increased concentration of 3-IAA and decreased tumour weight—were lost in NR-microbiota-colonized mice (Extended Data Fig. 2l,m). These data suggest that the effect of the tryptophan-high diet is mediated by 3-IAA, but we cannot rule out additional mechanisms contributing to the observed increased response in this set of experiments. Therefore, we directly supplemented 3-IAA in specific-pathogen-free (SPF) mice and found that our intervention was sufficient to reach the concentrations measured in mice that were colonized with R microbiota, and to increase the efficacy of chemotherapy not only in SPF but also in NR-colonized gnotobiotic mice (Fig. 1h and Extended Data Fig. 3a–c). We also tested other microbiota-modulated metabolites that have implications in different gastrointestinal cancers, such as the secondary bile acid deoxycholic acid (DCA) and the primary bile acid glycocholic acid (GCA)^18,19^. In addition, we chose to test two other metabolites that were increased in R patients or R-microbiota-colonized gnotobiotic mice—namely, the indole derivate indole-3-propionic acid (IPA) and hippuric acid—in combination with FIRINOX. None of these metabolites led to a similar efficacy to that of 3-IAA (Extended Data Fig. 3d,e). Together, these data indicate that the microbiota-derived tryptophan metabolite 3-IAA is increased in the serum of humans and mice that respond to chemotherapy, and that the levels of 3-IAA can be modulated by dietary interventions with tryptophan. Moreover, 3-IAA rendered even chemotherapy-resistant PDAC susceptible to the treatment.

## The effect of 3-IAA is licensed by myeloperoxidase

**Extended Data Fig. 4:**
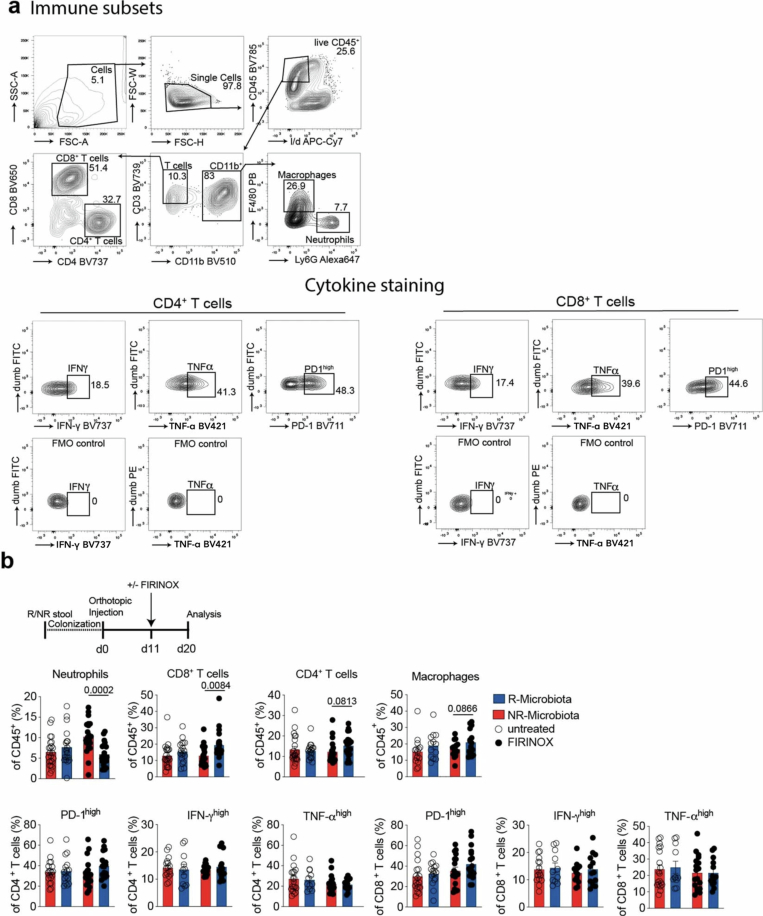
Tumour-infiltrating immune cells of KPC tumours from gnotobiotic mice. **a**, Flow cytometric gating strategy to classify immune subsets (left panel) or intracellular cytokines and coinhibitory receptors (right panel). Immune subsets were determined using the following marker combinations on live CD45^+^ immune cells: CD8^+^ T cells (CD11b^−^CD3^+^CD8^+^); CD4^+^ T cells (CD11b^−^CD3^+^CD4^+^); neutrophils (CD11b^+^Ly6G^+^); and macrophages (CD11b^+^Ly6G^−^F4/80^+^). **b**, Immune subsets or cytokine profiles and PD-1 expression of immune cells from orthotopic tumours obtained from gnotobiotic mice colonized with R or NR microbiota was analysed at day 20 of the experiment. Each symbol represents one mouse. Three to five independent experiments were pooled (n = 11 to 23 in total). Error bars indicate SEM, significant p-values are indicated and were determined by two-tailed t-test. Source data

**Extended Data Fig. 5:**
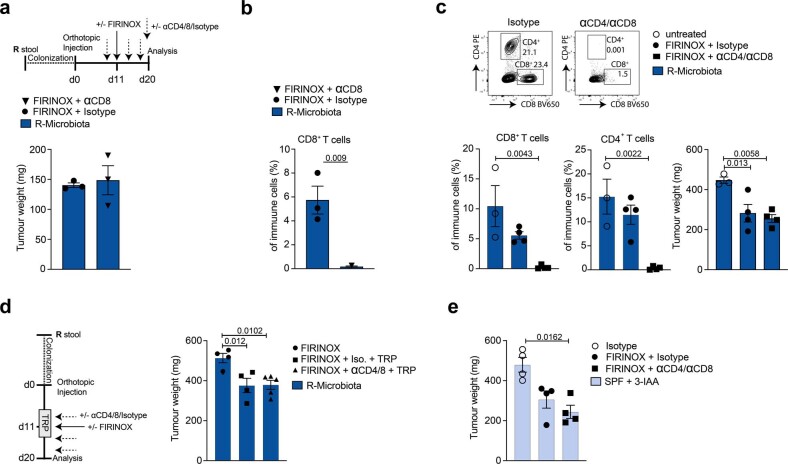
CD4^+^ and CD8^+^ T cells are dispensable for the efficacy of 3-IAA and FIRINOX. R-microbiota-colonized gnotobiotic mice were injected with KPC tumour cells orthotopically. Before treatment with FIRINOX, mice were injected with either isotype control antibody or CD8-depleting antibody every third day, as indicated. The tumour weight (**a**) and the depletion efficacy of intratumoral CD8^+^ T cells (**b**) as relative to total immune cells (CD45^+^), determined by flow cytometry, is shown at day 20 of the experiment (n = 3). **c**, As in **a**, except that CD4^+^ and CD8^+^ T cells were depleted simultaneously (n = 3 or 4). **d**, R-microbiota-colonized gnotobiotic mice with orthotopic KPC tumours were treated with FIRINOX, FIRINOX + tryptophan-high diet (d8–12) + isotype control antibody or FIRINOX + tryptophan-high diet (d8–12) + CD4/CD8-depleting antibody (n = 4 or 5). Tumour weight at day 20 of the experiment is depicted. **e**, As in **c**, except that SPF mice were treated with 3-IAA +/− FIRINOX and CD4/CD8-depleting or isotype control antibody (n = 4). Tumour weight is depicted at day 20 of the experiment. Each symbol represents one mouse. One experiment each was performed. Error bars indicate SEM, significant p-values are indicated and were determined by two-tailed t-test (**a**,**b**), one-way ANOVA followed by Dunnett’s (**c**,**d**) post-hoc test or Kruskal–Wallis test followed by Dunn’s post-hoc test (**e**). Source data

Microbiota-derived metabolites, especially tryptophan metabolites, have a crucial role in shaping innate and adaptive immunity^17^, which in turn have an important role in determining the efficacy of chemotherapy and prognosis in PDAC^11,20^. Therefore, we decided to analyse tumour-infiltrating immune cells in chemotherapy-naive or treated R- or NR-microbiota-colonized gnotobiotic mice. We consistently observed increased frequencies of CD8^+^ T cells and decreased neutrophils in R- compared to NR-microbiota-colonized mice after chemotherapy. We did not observe any changes in tumour-infiltrating immune cells when comparing untreated mice (Extended Data Fig. 4a,b). CD8^+^ T cells can induce the regression of PDAC when activated by the intestinal microbiota^11,21^. However, depleting CD8^+^ or both CD4^+^ and CD8^+^ T cells through antibody treatments did not decrease the efficacy of chemotherapy in R-microbiota-colonized mice that received either a standard or a high-tryptophan diet (Extended Data Fig. 5a–d). Similarly, depletion of CD4^+^ and CD8^+^ T cells did not lower the efficacy of 3-IAA and FIRINOX in SPF mice (Extended Data Fig. 5e). Thus, the effect of 3-IAA does not seem to depend on the presence of T cells.

**Fig. 2:**
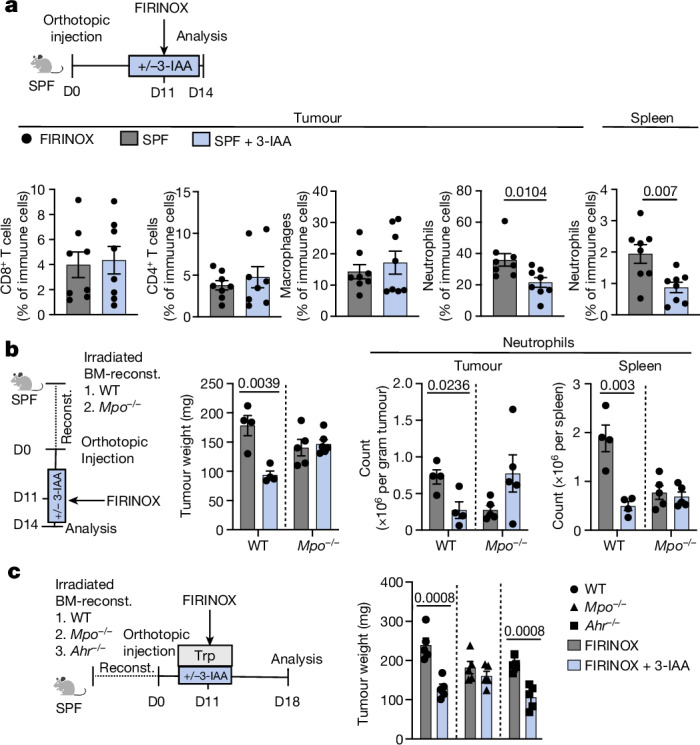
The efficacy of 3-IAA and FIRINOX is licensed by MPO. **a**, SPF mice were orthotopically injected with KPC cells and treated with FIRINOX with or without 3-IAA, and tumours were analysed at day three after FIRINOX treatment (*n* = 8 each). Immune subsets of orthotopic tumours or respective spleens were determined by flow cytometry. CD8^+^ T cells (CD3^+^CD8^+^); CD4^+^ T cells (CD3^+^CD4); macrophages (CD11b^+^F4/80^+^); neutrophils (CD11b^+^Ly6G^+^) are shown as relative to total living immune cells (CD45^+^). **b**, Tumour weight and counts of immune subsets of orthotopic KPC tumours or respective spleens of irradiated and wild-type (WT; *n* = 4) or *Mpo*^*–/–*^ (*n* = 5 or 6) bone-marrow (BM)-reconstituted mice treated and analysed as in **a**. **c**, Irradiated and wild-type, *Mpo*^*–/–*^ or *Ahr*^*–/–*^ bone-marrow-reconstituted mice received KPC cells orthotopically and were treated with FIRINOX or FIRINOX + 3-IAA for five days (*n* = 5 each). All mice received a four-day dietary intervention with a high-tryptophan diet. Tumour weight was assessed at day seven after FIRINOX treatment. Each symbol represents one mouse. Two independent experiments were pooled (**a**) or one out of two independent experiments (**b**,**c**) is shown. Error bars indicate s.e.m. Significant *P* values are indicated and were determined by two-tailed Mann–Whitney test (**a**) or two-tailed t-test (**b**,**c**). Source data

**Extended Data Fig. 6:**
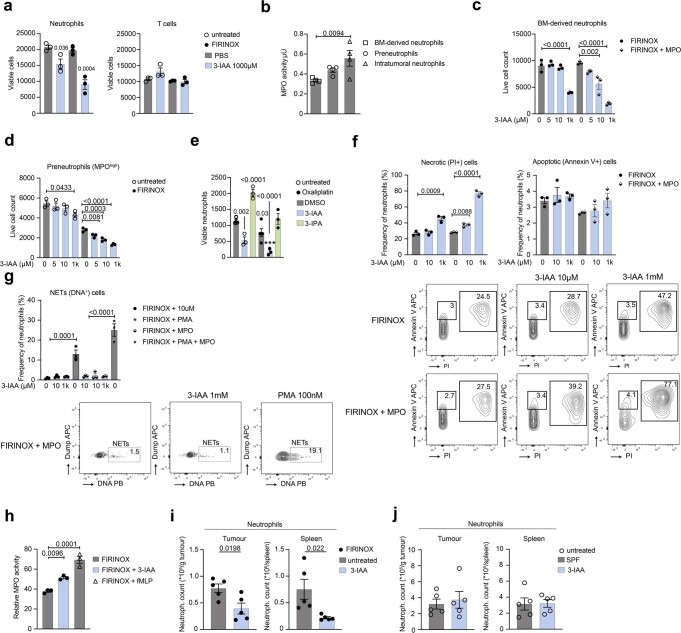
3-IAA and chemotherapy induce cell death in neutrophils. **a**, FACS-sorted T cells or neutrophils were cultured for 24 h +/− 1,000 μM 3-IAA and +/− FIRINOX (3.2 μM Oxaliplatin, 5.6 μM Irinotecan and 19.2 μM 5-FU). Viability was assessed via flow cytometry (n = 3, biological replicates). **b**, MPO activity was determined in 50,000 FACS-sorted bone-marrow-derived neutrophils, neutrophil precursors (lineage-negative, CD115^−^, Ly6B^+^, Ly6G^int-low^) from the bone marrow or PDAC-infiltrating neutrophils using a fluorometric MPO activity assay kit (n = 4, biological replicates). **c**, FACS-sorted bone-marrow-derived neutrophils were cultured in the presence of increasing dosages of 3-IAA +/− MPO +/− FIRINOX (3.2 μM Oxaliplatin, 5.6 μM Irinotecan and 19.2 μM 5-FU; n = 3, biological replicates). Cell numbers were quantified using flow cytometry after 48 h of culture. **d**, FACS-sorted neutrophil precursors were cultured in the presence of increasing dosages of 3-IAA +/− FIRINOX (3.2 μM Oxaliplatin, 5.6 μM Irinotecan and 19.2 μM 5-FU; n = 3, biological replicates). Cell numbers were quantified using flow cytometry after 48 h of culture. **e**, As in **a**, except only neutrophils were cultured in the presence of +/− Oxaliplatin (8 μM Oxaliplatin) and 1,000 μM 3-IAA, 1,000 μM 3-IPA or DMSO at similar concentrations (n = 3 to 6, biological replicates). **f**, As in **c**, except cell death of neutrophils was defined by flow cytometry using Annexin V and PI staining. Frequencies are shown relative to total neutrophils (n = 3, biological replicates). **g**, As in **c**, except NET formation was defined by SYTOX DNA staining after three hours of treatment (n = 3, biological replicates). **h**, Degranulation of 1 × 10^6^ FACS-sorted bone-marrow-derived neutrophils was measured after 30 min of stimulation with FIRINOX (3.2 μM Oxaliplatin, 5.6 μM Irinotecan and 19.2 μM 5-FU), 1 mM 3-IAA + FIRINOX or FIRINOX + 1 μM fMLP using an MPO activity assay kit (*n* = 3, biological replicates). **i**, KPC tumour cells were orthotopically injected into SPF mice and mice were treated +/− 500 mg/kg 3-IAA for 5 consecutive days and FIRINOX at day 11 (n = 5 each). Immune cells from the spleen and tumour were analysed by flow cytometry at day 3 after FIRINOX treatment. Neutrophil counts in the spleen or tumour are shown. **j**, As in **i**, except only 3-IAA, but no FIRINOX was applied (n = 5 each). Each symbol represents one mouse or one in vitro replicate. One experiment (**b**) or one out of three (**c**,**d**) or two (**a**,**e**–**j**) independent experiments are shown. Error bars indicate SEM, significant p-values are indicated and were determined by one-way ANOVA followed by Dunnett’s (**a**–**h**) post-hoc test or two-tailed t-test (**i**,**j**). Source data

Neutrophils are highly abundant in PDAC, and a low neutrophil count (neutropenia) is associated with a good prognosis in mPDAC^22^. 3-IAA is specifically toxic to cells with a high concentration of myeloperoxidase (MPO), which is a hallmark of neutrophils^23^. Mechanistically, MPO can oxidize 3-IAA, inducing toxic products such as 3-methylene-2-oxindole (MOI)^24,25^. In line with this, we found that culturing bone-marrow-derived neutrophils, but not T cells (neutrophils have higher levels of MPO than T cells), with 3-IAA and FIRINOX led to reduced cell survival (Extended Data Fig. 6a). Moreover, adding MPO to bone-marrow-derived neutrophil cultures or using immature neutrophils (pre-neutrophils) that have higher levels of endogenous MPO (ref. ^26^ and Extended Data Fig. 6b) increased the toxicity of 3-IAA and FIRINOX (Extended Data Fig. 6c,d). By contrast, IPA did not increase the efficacy of FIRINOX in inducing neutrophil cell death in vitro (Extended Data Fig. 6e). Further characterization of the response of neutrophils to 3-IAA and FIRINOX revealed that the treatment induced neutrophil degranulation, which was measured as the release of MPO, and necrosis, but did not lead to the formation of neutrophil extracellular traps (NETs) or apoptosis (Extended Data Fig. 6f–h). Next, we wondered whether these results were also reproducible in vivo. The combination of 3-IAA and FIRINOX reduced the frequency and number of neutrophils in the tumour and spleen of SPF mice (Fig. 2a and Extended Data Fig. 6i). Notably, this was not the case for 3-IAA treatment alone (Extended Data Fig. 6j).

**Extended Data Fig. 7:**
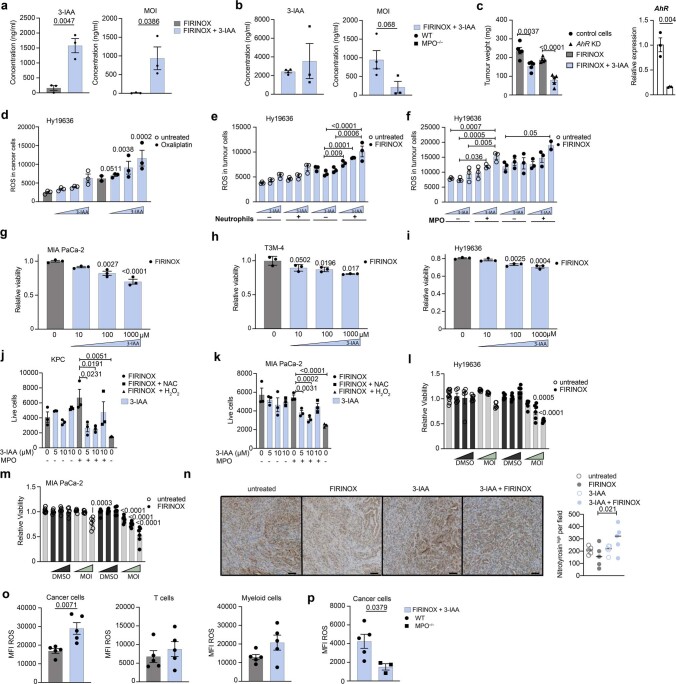
3-IAA and MPO increase ROS and decrease cell viability. **a**, Concentrations of 3-IAA measured using LC–MS/MS (left) or MOI (right) of tumours isolated from SPF mice treated with FIRINOX +/− 3-IAA five hours after treatment (n = 3 biological replicates). **b**, Concentrations of 3-IAA (left) or MOI (right) were measured in tumours isolated from WT or *Mpo*^*–/–*^ bone-marrow-reconstituted mice treated with FIRINOX + 3-IAA using LC–MS/MS five hours after treatment (n = 3 or 4 biological replicates). **c**, SPF mice received KPC control or *Ahr* KD cells orthotopically and were treated with FIRINOX (d11) or FIRINOX + 3-IAA (d9–13) (n = 4 or 5). Tumour weight was assessed at day 20 of the experiment. **d**, KPC tumour cells were cultured in the presence of increasing dosages of 3-IAA +/− Oxaliplatin (8 μM Oxaliplatin; n = 2-3 biological replicates). ROS expression was determined via flow cytometry using CellROX dye. Hy19636 cells were cultured with increasing dosages of 3-IAA +/− 5 × 10^4^ neutrophils (**e**) or MPO 200mU/ml (**f**) and +/− FIRINOX (1.6 μM Oxaliplatin, 2.8 μM Irinotecan and 9.6 μM 5-FU; n = 2-3 biological replicates). ROS expression was assessed via flow cytometry. Only p-values for relevant groups are shown for clarity. MIA PaCa-2 (**g**), T3M-4 (**h**) or Hy19636 (**i**) cells were cultured with increasing dosages of 3-IAA +/− FIRINOX (3.2 μM Oxaliplatin, 5.6 μM Irinotecan and 19.2 μM 5-FU) for 6 h (n = 3 biological replicates). Viability was assessed by MTS/MTT assay and displayed as relative to untreated cells after 48 h. KPC (**j**) or MIA PaCa-2 (**k**) cells were cultured in the presence of increasing dosages of 3-IAA +/− MPO 400 mU/ml +/− FIRINOX (3.2 μM Oxaliplatin, 5.6 μM Irinotecan and 19.2 μM 5-FU; n = 3 biological replicates). Cell numbers were quantified using flow cytometry after 48 h of culture. Hy19636 (**l**) or MIA PaCa-2 (**m**) cells were cultured with increasing dosages of MOI (100, 200 or 400 μM) +/− FIRINOX (1.6 μM Oxaliplatin, 2.8 μM Irinotecan and 9.6 μM 5-FU; n = 5 to 12 biological replicates). Viability was assessed using the MTS assay and displayed as relative to untreated cells after 24 h. **n**, KPC tumour cells were orthotopically injected into SPF mice and mice were left untreated or treated with either FIRINOX (d11), 3-IAA (d9–13) or 3-IAA and FIRINOX (n = 5 each). Statistics and representative pictures show orthotopic tumours three days after indicated treatment with staining for nitrotyrosine (ROS). **o**, SPF mice were injected with orthotopic tumours and were treated with FIRINOX or FIRINOX + 3-IAA (n = 5 each). After five hours of treatment, ROS accumulation was analysed using flow cytometry. ROS levels of cancer cells (Epcam^+^), T cells (CD45^+^CD3^+^) or myeloid cells (CD45^+^CD11b^+^) are shown. **p**, Irradiated and WT (n = 5) or *Mpo*^*–/–*^ (n = 3) bone-marrow-reconstituted mice received KPC cells orthotopically and were treated with FIRINOX or FIRINOX + 3-IAA as in **o**. ROS levels were assessed by flow cytometry as in **o**. One (**a**,**b**,**n**) or one out of three (**d**,**g**–**i**) or two (**c**,**e**,**f**,**j**–**m**,**o**,**p**) independent experiments are shown. Error bars indicate SEM or median (**n**), significant p-values are indicated and were determined by two-tailed t-test (**a**–**c**,**o**,**p**), one-way ANOVA followed by Dunnett’s (**d**,**g**–**n**) or Tukey’s (**e**,**f**) post-hoc test. Source data

Given the crucial role of neutrophil-derived MPO in the release of toxic 3-IAA products, we wondered whether this could explain the increased efficacy of 3-IAA and FIRINOX on tumour growth. To address this question, we established bone marrow chimeras from either wild-type or *Mpo*^*–/–*^ mice. As expected, treatment with FIRINOX and 3-IAA resulted in the depletion of neutrophils and showed synergistic efficacy in wild-type reconstituted mice (Fig. 2b). However, in mice that were reconstituted with *Mpo*^*–/–*^ bone marrow, the addition of 3-IAA did not result in smaller tumours (Fig. 2b), suggesting that MPO is essential for the efficacy of 3-IAA and FIRINOX. Further supporting the role of MPO, the 3-IAA oxidation product MOI was increased in tumours from wild-type SPF mice after treatment with 3-IAA and FIRINOX compared to that with FIRINOX alone (Extended Data Fig. 7a). In addition, the levels of MOI were strongly reduced in tumours from *Mpo*^*–/–*^ bone-marrow-reconstituted mice (Extended Data Fig. 7b).

Finally, considering the key role of the aryl hydrocarbon receptor (AhR*)* as an intracellular receptor for 3-IAA and other indols^27^, we wondered whether *Ahr*^*–/–*^ bone-marrow-reconstituted mice are resistant to 3-IAA and FIRINOX. The size of the tumours in *Ahr*^*–/–*^ bone-marrow-reconstituted mice was reduced to the same extent as that of tumours from wild-type reconstituted mice after treatment with 3-IAA and FIRINOX (Fig. 2c), arguing against a role of the AhR in mediating the efficacy of 3-IAA and FIRINOX treatment. Moreover, the AhR was also dispensable in cancer cells as *Ahr*-knockdown KPC cells responded to 3-IAA and FIRINOX treatment in vivo (Extended Data Fig. 7c).

These data show that the efficacy of 3-IAA and FIRINOX is licensed by immune cell-derived MPO, but not by AhR signalling.

## The effect of 3-IAA depends on ROS and autophagy

Oxidation of 3-IAA via MPO induces ROS in cultured neutrophils^23^ and ROS are major mediators of chemotherapy-induced cell death^28^. Therefore, we hypothesized that the efficacy of 3-IAA and FIRINOX in the presence of MPO is mediated by ROS. To address this, we cultured mouse and human PDAC cells with increasing dosages of 3-IAA, 3-IAA and neutrophils or 3-IAA and MPO with or without chemotherapy. 3-IAA directly increased ROS in a dose-dependent manner. The addition of neutrophils or MPO further enhanced the accumulation of ROS in cancer cells, and the addition of MPO reduced the viability of mouse and human PDAC cell lines (Extended Data Fig. 7d–k). Notably, direct addition of the 3-IAA oxidation product MOI also increased the efficacy of FIRINOX in mouse and human PDAC cells (Extended Data Fig. 7l,m).

Furthermore, to test the effects of 3-IAA and FIRINOX treatment on ROS induction in vivo, we treated SPF mice with either 3-IAA and FIRINOX or FIRINOX alone. In line with our in vitro data, treatment with 3-IAA and FIRINOX induced high oxidative stress, measured as ROS in cancer cells by flow cytometry or as nitrotyrosine in whole-tumour histology stains (Extended Data Fig. 7n,o). Further strengthening the link between ROS production and the oxidation of 3-IAA, we observed much lower levels of ROS after treatment with 3-IAA and FIRINOX in cancer cells from *Mpo*^*–/–*^ compared to wild-type bone-marrow-reconstituted mice, by flow cytometry (Extended Data Fig. 7p).

**Fig. 3:**
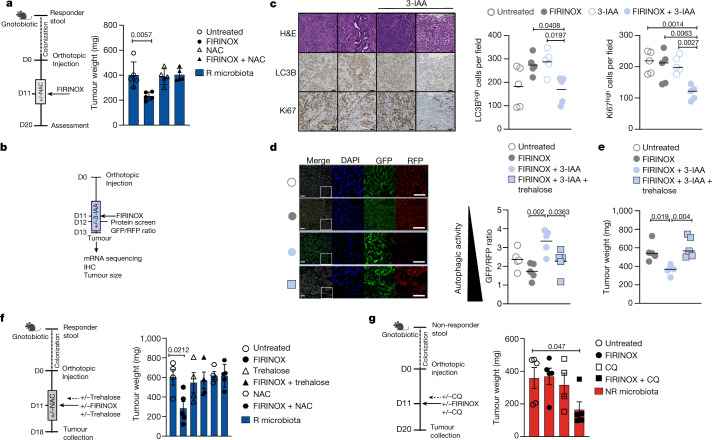
Treatment with 3-IAA and FIRINOX results in reduced autophagic activity. **a**, Gnotobiotic mice were colonized with R microbiota and KPC cells were orthotopically injected. Mice were untreated or treated with FIRINOX, NAC (day 9–13) or FIRINOX + NAC (*n* = 5 each). Tumour weight at day 20 of the experiment is shown. **b**, SPF mice bearing orthotopic KPC tumours were substituted +/− 3-IAA, treated with FIRINOX and analysed as indicated. IHC, immunohistochemistry. **c**, Representative images of orthotopic tumours stained with haematoxylin and eosin (H&E), LC3B or Ki67 (left) and respective statistics for positive cells per field (*n* = 5 each) (right). Scale bars, 50 μm. **d**,**e**, The GFP-LC3B-RFP reporter cell line Hy19636_GLRM was injected into SPF mice and mice were treated as indicated (*n* = 5 each). The graphs show the GFP/RFP ratio at day one (**d**) and tumour weight at day three (**e**) after FIRINOX treatment. Representative merged immunofluorescence images or indicated areas with a magnification of 3× are shown; scale bars, 10 μm. **f**, KPC cancer cells were orthotopically injected into R-microbiota-colonized mice and the indicated treatment was applied as shown in the scheme (*n* = 4 or 5). Tumour weight is shown at day 18 of the experiment. **g**, Tumour weight of orthotopic KPC tumours from gnotobiotic mice colonized with NR microbiota is shown nine days after the indicated treatment (*n* = 4 or 5). CQ, hydroxychloroquine. One experiment (**c**) or one out of two independent experiments (**a**,**d**,**e**–**g**) is shown. Each symbol represents one mouse. Error bars indicate s.e.m. Significant *P* values are indicated and were determined by one-way ANOVA followed by Dunnett’s (**a**,**c**,**f**) or Tukey’s (**d**,**e**) post-hoc test or Kruskal–Wallis test followed by Dunn’s post-hoc test (**g**). Source data

**Extended Data Fig. 8:**
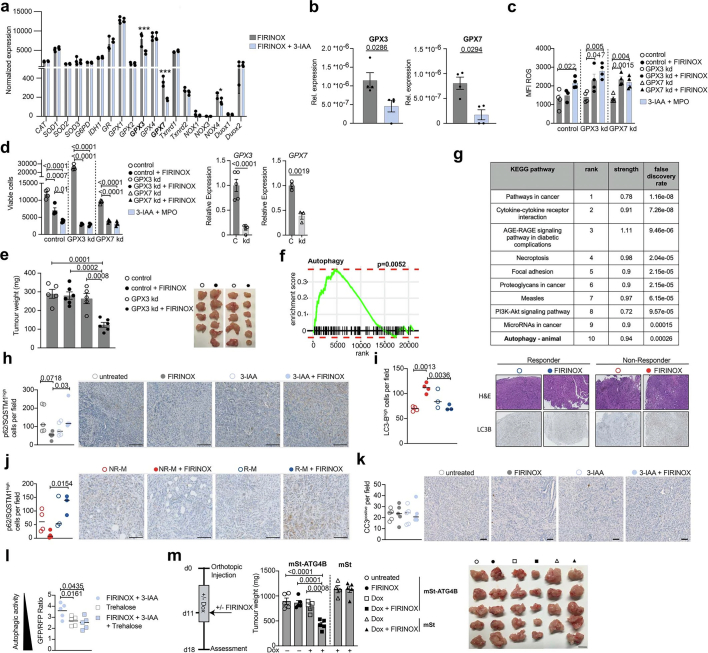
3-IAA and FIRINOX treatment decreases autophagy in vivo. **a**, Normalized expression of ROS-producing or degrading enzymes found in mRNA sequencing data of tumours obtained from mice treated with FIRINOX compared to tumours treated with FIRINOX + 3-IAA (n = 3 biological replicates each; p values: GPX3 (0.00012), GPX7 (1.2*10^−8^), NOX4 (0.023)). **b**, KPC tumour cells were cultured in the presence of FIRINOX (3.2 μM Oxaliplatin, 5.6 μM Irinotecan and 19.2 μM 5-FU) +/− 10 μM of 3-IAA and 400 mU/ml MPO (n = 4 biological replicates). RNA expression of GPX 3 and 7 was measured using qPCR after 24 h of treatment. Expression is depicted as relative to a housekeeping gene. **c**, Scramble control-transfected cells, *Gpx3* or *Gpx7* knockdown cells were treated with +/− FIRINOX (3.2 μM Oxaliplatin, 5.6 μM Irinotecan and 19.2 μM 5-FU) +/− 10 μM of 3-IAA and MPO and ROS was assessed using flow cytometry (n = 3 or 4 biological replicates). **d**, as in **c**, except cell numbers were calculated using flow cytometry (n = 4 biological replicates). **e**, SPF mice were injected with orthotopic tumours using scramble control or GPX3 KD cells and were treated with +/− FIRINOX at day eleven of the experiment (n = 5 or 6). Tumour size was measured at day eight after FIRINOX treatment. **f**, GSEA enrichment plot depicting positive enrichment of reactome pathway autophagy in total tumours obtained from mice treated with FIRINOX compared to tumours treated with FIRINOX and 3-IAA (n = 3 biological replicates). **g**, KPC tumour cells were orthotopically injected into SPF mice and mice were treated with either 3-IAA and FIRINOX or FIRINOX alone (n = 3 each). One day after FIRINOX treatment, intratumoral proteins were analysed and compared. Proteins downregulated in tumours from 3-IAA and FIRINOX-treated mice were analysed for enriched KEGG pathways. The top 10 pathways are depicted and the full list of proteins and up- or downregulated pathways is provided in SI tables 1 to 3. **h**, KPC tumour cells were orthotopically injected into SPF mice. Mice were treated +/− FIRINOX (d11), +/− 3-IAA (d9–13) and p62/SQSTM1 staining was analysed at day three after treatment. Statistics show the number of p62/SQSTM1^high^ cells per field (n = 5 each). Representative pictures are shown. The scale bar represents 100 μm. **i**,**j**, KPC tumour cells were orthotopically injected into gnotobiotic mice colonized with R or NR microbiota (n = 3 or 4). Mice were treated +/− FIRINOX and LC3-l/ll (**i**) or p62/SQSTM1 (**j**) staining was analysed at day 20 of the experiment. Statistics show the number of LC3-l/ll^high^ or p62/SQSTM1^high^ cells per field (n = 4). Tumours were pooled from three individual experiments with different donors each. Representative pictures are shown. The scale bar represents 100 μm. **k**, KPC tumour cells were orthotopically injected into SPF mice and treated and analysed as in **h**, except that staining for cleaved caspase-3 (CC3, apoptosis) was applied. The scale bar represents 50 μm. **l**, SPF mice were orthotopically injected with GFP-LC3B-RFP reporter cells and treated with FIRINOX + 3-IAA, trehalose or FIRINOX + 3-IAA + trehalose (n = 5 each). Tumours were analysed for GFP/RFP ratios via bright field imaging. The GFP/RFP ratio is shown for each respective group. **m**, SPF mice were injected with (mST-ATG4B) or mSt control cells. Mice received either doxycycline treatment via diet for seven days (d5 to d12) or standard food and were treated +/− FIRINOX (n = 4 or 5 each). Tumour size at day 18 of the experiment is depicted. Representative pictures show tumours at the end of the experiment. Scale bar shows one centimetre. One (**a**,**f**–**k**) or one out of two independent experiments (**b**–**e**,**l**) or three independent experiments with different lengths of doxycycline treatment (**m**) are shown. Each symbol represents one mouse or one *in vitro* replicate. Bars indicate SEM (**a**–**e**,**m**) or median (**h**–**l**), significant p-values are indicated and were determined as indicated in the methods section (**a**,**f**,**g**), by two-tailed Mann–Whitney test (**b**) one-way ANOVA followed by Dunnett’s (**c**, **l**) or Tukey’s (**d**, **e**, **i**, **j**, **m**) post-hoc test or by Kruskal–Wallis test followed by Dunn’s (**h**,**k**) post-hoc test. Source data

We then wondered which oxidative-stress-related pathways are involved in the accumulation of ROS in 3-IAA- and FIRINOX-treated tumours. To this end, we analysed the expression of known ROS-producing or ROS-degrading enzymes in mRNA sequencing data, comparing tumours from mice that were treated with 3-IAA and FIRINOX to tumours from mice treated with FIRINOX alone (Extended Data Fig. 8a). We found that the ROS-degrading enzymes glutathione peroxidase 3 (GPX3) and glutathione peroxidase 7 (GPX7) were downregulated in vivo, and treatment with 3-IAA, FIRINOX and MPO led to a similar downregulation of GPX3 and GPX7 in KPC cells in vitro (Extended Data Fig. 8b). Knockdown of *Gpx3* and *Gpx7* in cancer cells was sufficient to increase the accumulation of ROS after treatment with FIRINOX, and increased the susceptibility of the cancer cells to FIRINOX to a similar extent as 3-IAA and FIRINOX (Extended Data Fig. 8c,d). Of note, knockdown of *Gpx3* was sufficient to establish susceptibility to FIRINOX in vivo (Extended Data Fig. 8e). Finally, we could show that ROS accumulation is essential for the efficacy of 3-IAA and FIRINOX, because treatment with the ROS scavenger *N*-acetylcysteine (NAC) abolished the efficacy of FIRINOX in R-microbiota-colonized (that is, high-3-IAA) mice (Fig. 3a).

Next, we wondered what the molecular response to high levels of ROS is. To address this, we performed a targeted protein screen and mRNA sequencing on tumours isolated from SPF mice that were treated with 3-IAA and FIRINOX or FIRINOX alone (Fig. 3b). Autophagy was one of the main pathways that was downregulated in tumours isolated from mice treated with 3-IAA and FIRINOX (Extended Data Fig. 8f,g and Supplementary Tables 1–3). Notably, autophagy is an essential metabolic program for PDAC to proliferate and thrive^29^. Next, we found that the autophagy substrate LC3B was reduced and p62/SQSTM was increased in the tumours isolated from SPF mice that were treated with 3-IAA and FIRINOX (Fig. 3c and Extended Data Fig. 8h). In line with this, tumours from R-microbiota-colonized mice (high 3-IAA) also showed a reduced abundance of LC3B and an increased abundance of p62/SQSTM1 compared to tumours from NR-colonized mice (low 3-IAA) (Extended Data Fig. 8i,j). Changes in LC3B and p62/SQSTM were accompanied by a decline in tumour cell proliferation, measured as the expression of Ki67, in mice treated with 3-IAA and FIRINOX (Fig. 3c). However, we did not observe a change in the number of apoptotic cells, as measured by cleaved caspase-3 (Extended Data Fig. 8k), as late as three days after treatment. To test whether downregulation of autophagy is an essential downstream mechanism that explains the synergy of 3-IAA and FIRINOX, or whether it is merely associated with the observed reduction in cancer cell proliferation, we set up gain- and loss-of-function experiments in vivo. First, we observed that treatment with the disaccharide trehalose^30^ normalizes autophagic activity, as measured by a decreasing GFP/RFP ratio in autophagy reporter cells^31^ (Fig. 3d and Extended Data Fig. 8l). Second, trehalose was sufficient to completely reverse the treatment efficacy of 3-IAA and FIRINOX, similar to the ROS scavenger NAC (Fig. 3e,f). Third, treatment with the autophagy blocker hydroxychloroquine sensitized tumours from NR- microbiota-colonized mice (low 3-IAA) to FIRINOX treatment (Fig. 3g). Finally, inhibiting autophagy in cancer cells through a doxycycline-inducible dominant-negative ATG4B protein (mSt-ATG4B)^29^ increased the susceptibility to FIRINOX treatment in SPF mice in comparison to control cells (mSt) or mSt-ATG4B cells without doxycycline treatment (Extended Data Fig. 8m).

Overall, these data suggest that when 3-IAA and MPO are present during FIRINOX treatment, the accumulation of ROS increases and the stress adaptation of cancer cells is impaired (that is, autophagy is downregulated), ultimately inducing the reduced proliferation of tumour cells.

## 3-IAA has therapeutic potential

**Fig. 4:**
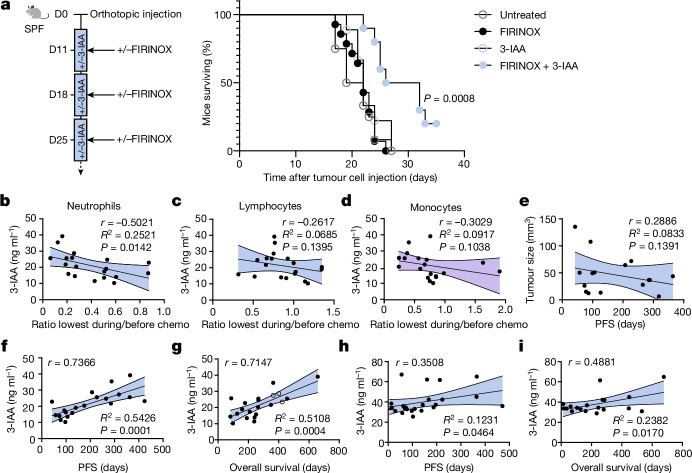
3-IAA is clinically relevant in PDAC. **a**, SPF mice were orthotopically injected with Hy19636 cells, treated as indicated and their overall survival is depicted in the Kaplan-Meier estimator (untreated *n* = 12; FIRINOX *n* = 14; 3-IAA *n* = 9; 3-IAA + FIRINOX *n* = 10). **b**–**d**, The 3-IAA serum concentration of patients from the Hamburg cohort was measured by chemiluminescence immune assay (CLIA) and correlated with the ratio of blood neutrophil (**b**), lymphocyte (**c**) or monocyte (**d**) counts at the time point of lowest overall leukocyte count (within the first three months of chemotherapy) and counts before start of chemotherapy. **e**, Tumour size as measured in CT scans was correlated with PFS in patients from the Hamburg cohort. **f**,**g**, 3-IAA serum concentration after two to three chemotherapy cycles of patients from the Hamburg cohort was correlated with PFS (**f**) or overall survival (**g**). One patient was excluded from **f**, because the patient’s cancer did not progress before the event of death. Patients represented with open circles are still alive and therefore excluded from the correlative analysis in **g**. **h**,**i**, The 3-IAA serum concentration before the start of treatment of patients from the Munich cohort was correlated with PFS (**h**) or overall survival (**i**). Each symbol represents one patient. Two independent experiments were pooled (**a**). Mean and 95% confidence intervals. *P* values are indicated and were determined by log-rank Mantel–Cox test (**a**) or simple linear regression and Pearson’s *r* (**b**–**i**). Source data

**Extended Data Fig. 9:**
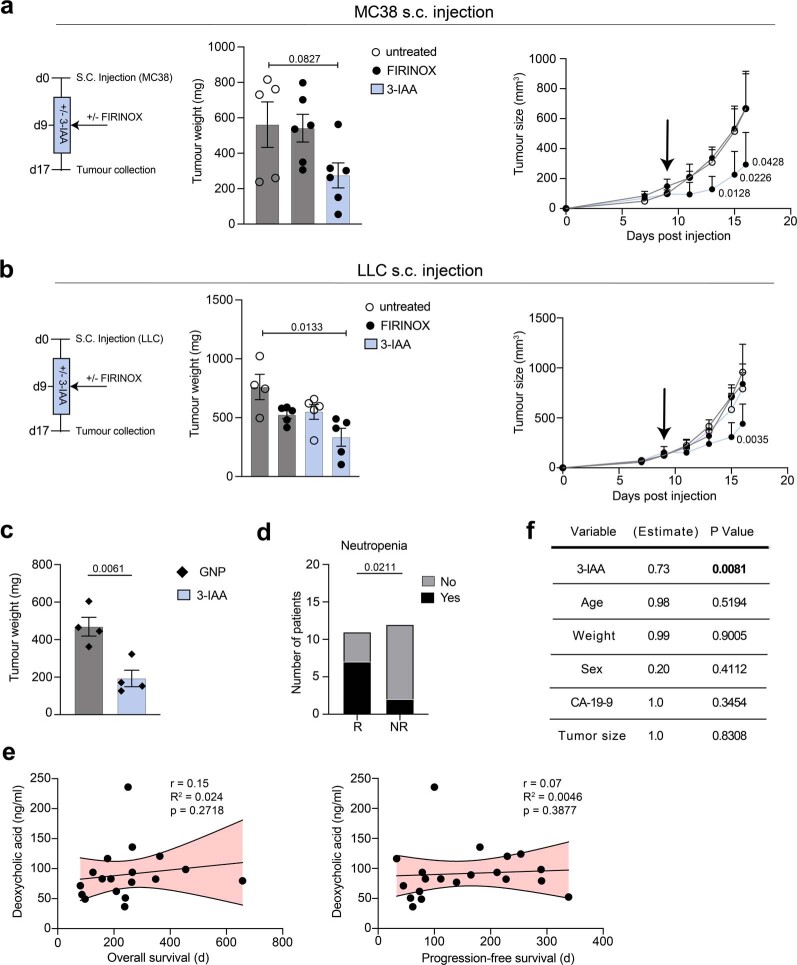
A 3-IAA and chemotherapy combination is effective in the treatment of colorectal and lung cancer cell lines. **a**,**b**, The MC38 colorectal cancer cell line (n = 5 or 6; **a**) or LLC lung cancer cell line (n = 4 or 5; **b**) was subcutaneously injected and mice were treated +/− FIRINOX and +/− 3-IAA (500 mg/kg) orally. Tumours were measured every other day and tumour weight was scaled to day 17 of experiment. **c**, Orthotopic KPC tumours were established and SPF mice were treated with GNP once +/− 3-IAA (500 mg/kg; n = 4 each). Tumour weight was assessed seven days after chemotherapy (d18). **d**, Statistics show the event of neutropenia (<1,5*10^9^/l) during the first 6 cycles of chemotherapy in patients from the Hamburg cohort responding or not responding to the chemotherapy (n = 11 R and 12 NR). **e**, DCA concentration was measured after two to three chemotherapy cycles in the serum of patients from the Hamburg cohort and was correlated with PFS (n = 20) or overall survival (n = 18). Each symbol represents one patient. **f**, Univariate Cox proportional hazard models were used to determine the effects of indicated variables on PFS in the Hamburg cohort. One experiment (**b**) or one out of two individual experiments (**a**) is shown. Error bars indicate SEM, significant p-values are indicated and were determined by one-way ANOVA followed by Dunnett’s post-hoc test (**a**, left statistic), Kruskal–Wallis test followed by Dunn’s (**b**, left statistic) post-hoc test, mixed-effects analysis followed by Dunnett’s post-hoc test (**a** and **b**, right statistic), two-tailed t-test (**c**), two-sided chi-square test (**d**), simple linear regression and Pearson’s *r* (**e**) or Cox regression (**f**). Source data

To further investigate potential therapeutic implications of 3-IAA, we tested the effect of repetitive applications of 3-IAA and FIRINOX on mouse survival, treatment of other cancer entities and the efficacy of 3-IAA with a different chemotherapy combination, namely GnP. Up to three cycles of 3-IAA and FIRINOX together—but neither alone—significantly increased the duration of survival with orthotopic PDAC (Fig. 4a). Notably, 3-IAA was also synergistic with FIRINOX in the treatment of subcutaneous colorectal (MC38) or lung (LLC) tumours (Extended Data Fig. 9a,b) and also synergized with GnP in orthotopic PDAC (Extended Data Fig. 9c). These findings highlight the potential general role of 3-IAA in cancer treatment.

Finally, we wanted to assess the relevance of our findings in humans. We observed higher rates of neutropenia in patients who responded to chemotherapy, consistent with our mouse data and the known association between neutropenia and PDAC prognosis^22^ (Extended Data Fig. 9d). Notably, the levels of neutrophil decrease after chemotherapy, but not lymphocyte or monocyte decrease, correlated with 3-IAA serum concentrations, as expected from their different levels of MPO (Fig. 4b–d). Furthermore, we observed a significant correlation of the 3-IAA serum concentration and PFS or overall survival in our observation cohort (Hamburg cohort; Extended Data Table 1), which was absent for the secondary bile acid DCA (Fig. 4f,g and Extended Data Fig. 9e). We validated these findings in a second cohort of patients with mPDAC from the Ludwig-Maximilians-University (LMU) Hospital (Munich cohort; Extended Data Table 1) (Fig. 4h,i). Reflecting the missing value of other clinically assessable markers to predict a chemotherapy response a priori, neither patient-specific (age, gender and weight) nor tumour-specific (tumour size and tumour marker) variables were significantly associated with PFS (Fig. 4e and Extended Data Fig. 9f).

## Discussion

In summary, our results identify the microbiota-derived metabolite 3-IAA as a key amplifier of the response to chemotherapy in PDAC. When 3-IAA and MPO are present in high concentrations during FIRINOX treatment, the accumulation of ROS increases and the stress adaptation of cancer cells is impaired, which ultimately results in the reduced proliferation of PDAC cells. Despite the limited number of patients analysed (Hamburg cohort *n* = 23, of whom 21 had available serum samples; Munich cohort *n* = 24), we observed a robust correlation between 3-IAA serum concentrations measured during chemotherapy (Hamburg cohort) or even before the start of chemotherapy (Munich cohort) and PFS or overall survival.

These data set the early premise to initiate clinical trials that aim to raise the serum concentration of 3-IAA during chemotherapy treatment through direct treatment or dietary intervention, and ultimately even increase the survival of NR patients with PDAC. Increasing the 3-IAA substrate tryptophan through a specific diet could be easily achieved; however, this approach is affected by the composition of the microbiota, as suggested by the finding that 3-IAA serum concentrations only increased in R-microbiota-colonized mice after dietary intervention. Therefore, when it becomes possible to produce 3-IAA under good manufacturing practice standard conditions, direct treatment with 3-IAA would be ideal, especially considering its capacity to bypass the presence of an undesired microbiota, as shown by our experiments in mice colonized with a NR microbiota.

Before planning future clinical trials, it is important to consider that, in the absence of chemotherapy, indoles or other tryptophan metabolites can impair the development of anti-tumour immune responses through the AhR in PDAC^16^. However, our data show that four to five days of 3-IAA treatment without chemotherapy is not sufficient to affect the growth of the tumour, and only reduces the growth of the tumours via an AhR-independent mechanism when it is combined with chemotherapy. Nevertheless, more observational studies are needed before proceeding with any interventional ones.

Alternatively, faecal microbiota transplantation, which is being tested for the treatment of patients with cancer^8,9^, may be sufficient to increase the serum concentrations of 3-IAA and, accordingly, the response to chemotherapy, as shown in our gnotobiotic mice experiments using the stool of ten different human donors. Our study suggests that *B. fragilis* and *B. thetaiotaomicron* are enriched in the microbiota of R patients and are able to produce 3-IAA. However, larger studies are needed to reveal whether these bacteria are indeed responsible for the production of 3-IAA production in R patients, considering the vast number of taxonomically distinct bacterial species that are able to produce 3-IAA (refs. ^14,15^). Such studies are of interest to narrow the selection of donors for potential future studies of faecal microbiota transplantation.

In parallel to the effect of tumour size through treatment with 3-IAA and FIRINOX, we also observed a depletion of neutrophils in mouse models of PDAC. In patients with PDAC, we observed a positive correlation between the depletion of neutrophils during chemotherapy and the serum concentration of 3-IAA in the Hamburg cohort. These data suggest that, as in mice, 3-IAA can also contribute to the development of neutropenia in humans. However, given that other factors can lead to neutropenia, such as an altered chemotherapeutic metabolism, blocked excretion of chemotherapeutics or differing chemotherapeutic dosages^32^, studies that focus on this particular aspect are needed to firmly conclude whether there is a causative link between 3-IAA and neutropenia in humans. Furthermore, as neutrophil levels are a key predictor of survival in many cancer types, investigations of the described mechanism in other cancers may be warranted^33,34^.

Our findings have the potential to be transformative for the treatment of PDAC and other cancer types such as colorectal cancer, especially for patients who are treated with FIRINOX-based regimens. In addition, our findings will trigger the development of studies that address the effects of microbiota-derived metabolites on the ROS–autophagy axis in response to chemotherapeutic treatments.

## Methods

### Patients and human material

Patients who had been diagnosed with mPDAC and scheduled for treatment with GnP or FOLFIRINOX were recruited to the study (*n* = 30). Informed consent was obtained from all patients as approved by the ethics commission Hamburg (Ethikkommission der Ärztekammer Hamburg, Germany). Patients who received antibiotics during the first three months of treatment, did not deliver pre-treatment stool, did not start chemotherapy or suffered from COVID-19 were excluded from the analyses (*n* = 7). Stool was collected before the start of chemotherapy using a home sampling kit (OMNIgene, Gut OMR-200) for shotgun metagenomic sequencing. One sample was excluded after sequencing owing to overloading of the sample tube that led to unsuccessful fixation and limited representation of the microbiota. Therefore, the final cohort for microbiota analysis contained 22 patients. The classification of R and NR patients was primarily based on tumour shrinkage of at least 25%, which is calculated as a decrease of the primary tumour and largest metastasis in CT scans comparing the time point before treatment initiation to the time point that revealed the best response during first-line treatment as assessed by the local radiologist (20 to 30% of changes are usually considered significant in CT response evaluation^35^). In cases of disease stabilization or missing CT scans, the criteria of a PFS of over 140 days (median PFS of real-world FOLFIRINOX and GnP cohorts^36^) or a decline of at least 40% in serum tumour markers (prognostic cut-off in palliative PDAC treatment^37^) were considered. Stool for functional experiments was collected in tubes without any additive, transported directly to the laboratory, subsequently diluted in 20% glycerol (Teknova, G1723), aliquoted and frozen at −80 °C. To preserve most of the donor microbiota for functional experiments, the maximum allowed turnaround time from toilet to freezer was 4 h. Blood was drawn at cycle three or four of chemotherapy treatment, mixed 1 to 1 with phosphate-buffered saline (PBS) and plasma was isolated using gradient centrifugation. Serum material of the Munich cohort was taken before the start of chemotherapy and processed according to local standards. Informed consent was obtained from all patients as approved by the ethics commission Munich (project number: 284-10).

### Animal models

All mice used in this study were of a C57BL/6 background. All mice were used in accordance with the institutional review board ‘Behörde für Soziales, Familie, Gesundheit und Verbraucherschutz’ (Hamburg, Germany). Mice were kept under SPF or germ-free conditions, with an ambient temperature of 20 ± 2 °C, humidity of 55 ± 10% and a dark–light cycle of 12 h. Age- and sex-matched littermates between 4 and 16 weeks old were used for the most part. *Mpo*^*–/–*^ bone marrow used to establish bone marrow chimeras was provided by S. Baldus and M. Mollenhauer. *Ahr*^*–/–*^ bone marrow used to establish bone marrow chimeras was provided by C. Esser.

For colonization of human microbiota, stool was thawed, washed with brain heart infusion broth (BHI) and diluted in BHI. Two hundred microlitres of the suspension was gavaged once orally to gnotobiotic mice housed in isocages. Two to four weeks later, tumour experiments were initiated.

Sample sizes were calculated on the basis of small pilot experiments and mice were randomized before the beginning of treatment. The person treating the mice was not blinded because of the complex treatment schedules. For models of orthotopic PDAC, 5 × 10^4^–10 × 10^4^ KPC, 2 × 10^5^ Hy19636, 1 × 10^5^ mSt-ATG4B/mSt or 1 × 10^5^ knockdown KPC (AhR, GPX, scramble control) cells were injected orthotopically in a 1:1 mixture of PBS and Matrigel (Corning, 356231). Tumour weight was assessed at day 20 after tumour cell injection unless otherwise indicated. For subcutaneous tumour growth, 2.5 × 10^5^ LLC-GFP or 1 × 10^6^ MC38 cells were subcutaneously injected into the flank. Subcutaneous tumour growth was measured every other day using a caliper. Tumour weight was assessed at day 17 after tumour cell injection. The maximum allowed diameter of subcutaneous tumours for active experimental mice was 1.5 cm and limits were not exceeded.

For bone marrow chimeras, recipient mice were irradiated with 9.6 Gy (BioBEAM 2000) 24 h before bone marrow transfer. One day later, 1 × 10^6^–4 × 10^6^ bone marrow cells isolated from wild-type, *Ahr*^*–/–*^ or *Mpo*^*–/–*^ mice were transferred by intravenous injection. Three to four weeks after transfer, cancer cells were orthotopically injected and treated as described below. Successful engraftment was validated by quantitative PCR (qPCR) on tumour-infiltrating immune cells.

Treatment with chemotherapy was initiated intraperitoneally (i.p.) at the indicated time point (usually day 11 after cancer cell injection). Oxaliplatin (Accord) 5 mg kg^−1^, irinotecan (Accord) 20 mg kg^−1^ and 5-FU (Medac) 50 mg kg^−1^ were used for FIRINOX treatment as described in other studies^38,39^. Folinic acid was not used and gemcitabine (Hexal) 120 mg kg^−1^ and nab-paclitaxel (Celgene) 30 mg kg^−1^ were used for GnP treatment. Depletion of T cells was achieved by treatment with 200 μg anti-CD8 antibody clone 53-6.7 (BioXcell, BE0004) alone or in combination with 200 μg anti-CD4 antibody clone GK1.5 (BioXcell, BE0003) every third day i.p., beginning one day before chemotherapy treatment. Control mice were treated with the respective isotype control clone 2A3 (BioXcell, BE0089) at similar concentrations and intervals. Autophagy induction was achieved using two i.p. injections of trehalose (Sigma, T9449) at a concentration of 3 g kg^−1^ one day before and on the day of chemotherapy treatment. Similarly, 60 mg kg^−1^ chloroquine (Sigma, C6628) dissolved in PBS was injected i.p. one day before and on the day of chemotherapy treatment.

Treatment with 3-IAA (500 mg kg^−1^) was applied by oral gavage using indole-acetic-acid sodium salt (Sigma, I5148) dissolved in PBS every day for five consecutive days (two days before chemotherapy until two days after chemotherapy), unless otherwise indicated. Indole-3-propionic acid (3-IPA; Sigma, 57400), GCA (Sigma, G2878), hippuric acid (Sigma, 112003) and DCA (Sigma, 30960) were dissolved in 1 M NaOH in PBS and pH-adjusted to 7.4 using 1 M HCl. The solution was gavaged for five consecutive days orally at a concentration of 500 mg kg^−1^ (3-IPA, hippuric acid) or 250 mg kg^−1^ each (GCA and DCA). NAC (Sigma, a7250) was applied for five days in the drinking water ad libitum at a concentration of 1 g l^−1^.

Dietary tryptophan modulation was initiated three days before chemotherapy until one day after treatment. In one experiment, tryptophan modulation was applied for a total of 14 days. Standard diet (2.3 g kg^−1^ tryptophan; Altromin, 1320) was changed to either synthetic crystalline AA tryptophan-free (0 g kg^−1^; SSNIFF, S9336-E701) diet or crystalline AA tryptophan-high (12 g kg^−1^; SSNIFF, S5714-E711) diet. Subsequently, the diet was changed back to the standard diet.

Dietary doxycycline (Sigma, D9891) was administered at a dosage of 625 mg kg^−1^ through the diet starting at day five or eight after cancer cell injection for a total of up to seven consecutive days. Subsequently, the diet was changed back to the standard diet.

For measurement of serum metabolites, blood was drawn at the end of the experiment or at the indicated time point. Blood was allowed to clot for 30 min and was centrifugated (1,000*g*) for 10 min thereafter. Serum was diluted and used as described in the specific section.

### DNA extraction and shotgun metagenomics

For DNA extraction, samples were isolated with the ZymoBIOMICS 96 MagBead DNA kit (Zymo Research, D4302) and purified with Zymo DNA Clean and Concentrator-5 (Zymo Research, D4004). Libraries were prepared with the NEBnext Ultra II DNA Library Prep Kit for Illumina (New England Biolabs, E7645) with 150 ng of total DNA, size selection of 400–500 bp and 4× PCR cycles.

For shotgun metagenomics, Illumina library preparation was performed using the NEBNext Ultra II FS DNA Library Prep Kit (New England Biolabs, E7805). The library preparation was performed according to the manufacturer’s instructions. The size selection was performed using AMPure XP beads (Beckman Coulter, A63882) and adaptor enrichment was performed using seven cycles of PCR using the NEBNext Multiplex oligos (New England Biolabs, E7335) from Illumina and then subjected to Illumina NovaSeq (2 × 150 bp) sequencing.

For bioinformatic analysis, raw reads were trimmed for low quality and filtered against the phix174 and human hg19 genome with bbduk (ref. ^40^). For taxonomic species profiling, all libraries were mapped against the Unified Human Gastrointestinal Genome collection (*n* = 4,644) using BBMap (refs. ^41,42^). Mapping rates were normalized into transcripts per million (TPM) and genomes with less than 10% genome coverage (genome breadth) were considered not prevalent in the sample. Data were summarized as metagenomics operational taxonomic units (OTUs) into biom format and analysed with phyloseq and LEfSe (refs. ^43,44^). Species-level functional profiling was performed with HUMAnN3 and also using the 9.9 million gene integrated reference catalogue of the human microbiome^41^.

### Bacterial strains and isolation

*Bacteroides thetaiotaomicron* (DSM 2079), *B. fragilis* (DSM 2151) and *Prevotella copri* (DSM 18205) were obtained from the German Collection of Microorganisms and Cell Cultures (DSMZ). For bacteria isolation, faecal samples frozen in glycerol were thawed and streaked out anaerobically in serial dilutions on BHI blood agar plates (5% defibrinated sheep’s blood & vitamin K_3_) supplemented with vancomycin. After growing the agar plates inside an incubator at 37 °C for 2 days, single colonies were picked into BHI-S medium in a 96-well plate and were further incubated for 24 h. The resulting bacterial cultures were screened by PCR using specific primers for *B. thetaiotaomicron* (*B. theta* F 5′-GAGGGTGTCGTATTTCCGAAGG-3′ R 5′-GTTCCCTGATCCAGTGTGTTGG-3′) or *B. fragilis* (*B. frag* F 5′-AATGATTCCGCATGGTTTCA-3′ R 5′-ATTTTGGGATTAGCATACGG-3′). After passaging PCR-positive wells on agar plates to obtain pure cultures and additional confirmation of identity by Sanger sequencing, resulting strains were maintained in BHI-S until further use. All bacterial strains were cultured and maintained anaerobically in BHI broth supplemented with 10% fetal bovine serum (FBS) and vitamin K_3_ (BHI-S).

### Extraction of bacterial supernatant

BHI-S medium supplemented with 1% tryptophan was inoculated from a fully grown overnight bacterial culture (1:50 ratio) and was incubated anaerobically until the early exponential phase. Cultures were taken out of the anaerobic chamber and were centrifuged at 4,700 rpm (4,816*g*) for 5 min at room temperature. The supernatant was removed and immediately frozen at −20 °C.

### 16S rRNA sequencing

DNA from tumours was extracted using the DNeasy Blood & Tissue Kit (Qiagen, 69504). Approximately 10 mg tissue was digested with Proteinase K in ATL buffer at 56 °C for 1 h. Afterwards, samples were processed according to the manufacturer’s protocol. Blank extraction controls were included during extraction of samples.

Variable regions V1 and V2 of the 16S rRNA gene were amplified using the primer pair 27F-338R in a dual-barcoding approach according to a previous report^45^. For tumours, 3.5 µl DNA was used for amplification and PCR products were verified using agarose gel electrophoresis. Final PCR products were normalized using the SequalPrep Normalization Plate Kit (Thermo Fisher Scientific, A1051001), pooled equimolarly and sequenced on the Illumina MiSeq v3 2×300bp (Illumina). Demultiplexing after sequencing was based on 0 mismatches in the barcode sequences. Data processing was performed using the DADA2^46^ workflow for big datasets (https://benjjneb.github.io/dada2/bigdata.html; the workflow adjusted for the V1–V2 region can be found here: https://github.com/mruehlemann/ikmb_amplicon_processing/blob/master/dada2_16S_workflow.R), resulting in abundance tables of amplicon sequence variants (ASVs). ASVs underwent taxonomic annotation using the Bayesian classifier provided in DADA2 and using the Ribosomal Database Project (RDP) version 16 release. Sequences that were not assignable to genus level were binned into the finest possible taxonomic classification.

### Metabolomic screen

We used ultra-high-pressure liquid chromatography–tandem mass spectrometry (UPLC–MS/MS) to acquire data in both positive and negative ionization modes that allowed for identification and quantification of 630 metabolites. Plasma samples were processed using the MxP Quant 500 Kit (Biocrates) according to the manufacturer’s instructions. In brief, 10 µl of plasma sample, calibration standard and control sample were transferred onto a filter containing internal standards for internal standard calibration. Filters were dried under a stream of nitrogen using a pressure manifold (Waters). Samples were incubated with derivatization reagent phenyl isocyanate for 60 min. After drying under nitrogen, analytes were extracted with 5 mmol l^−1^ ammonium acetate in methanol and the eluate was further diluted for the UPLC–MS/MS analysis. The targeted analysis covered 630 metabolites (https://biocrates.com/mxp-quant-500-kit/) detected by MS/MS after UPLC separation and flow injection analysis (FIA). Each measurement required two UPLC runs and three FIA runs to cover all metabolites. All analyses were performed on an ACQUITY UPLC I-Class system (Waters) coupled to a Xevo TQ-S mass spectrometer (Waters). Reversed-phase chromatographic separation was accomplished using a C18 LC-column (Biocrates) with 0.2% formic acid in water with 0.2% formic acid in acetonitrile as the eluent system. The FIA solvent was methanol, with a modifier provided by the kit manufacturer. Data analysis of the UPLC–MS/MS results was based on a seven-point curve or one-point calibration and internal standard normalization. Values below the lower threshold were set to zero. Concentration data were analysed using MetaboAnalyst v.5. Concentrations were log-transformed before analysis and raw *P* values and log_2_-transformed fold change values are shown in the graphics of Fig. 1.

### 3-IAA and DCA CLIA

To quantify 3-IAA or DCA serum concentrations, mouse or human serum was diluted 1 to 10 with PBS and the chemiluminescence immune assay (CLIA) (Abbexa, 3-IAA abx190011; DCA 258844) was performed according to the manufacturer’s protocol. For detection of 3-IAA in cultures, supernatant was processed as described above and directly used for the assay. Chemiluminescence was detected using FLUOstar Omega (BMG Labtech) with 1-s sampling per well, and a gain of 3,400 to 4,000 was individually adjusted for each assay. The concentration was determined using supplied standards and interpolated using Prism 8.4.0.

### 3-IAA and MOI measurement in tumour tissue

Frozen tumour tissue was mixed with ice-cold methanol 1:1–3 and homogenized using 0.4-mm beads at 5,500 rpm for 2 × 30 s using a homogenizer (Precyllys 24 touch). Extracts were centrifuged (10,000*g*) at 4 °C and the supernatant was used for LC–MS/MS analysis. Reversed-phase chromatography was conducted using a biphenyl stationary phase (Raptor Biphenyl (Restek), dimensions: 50 mm × 2.1 mm ID; particle size: 2.7 µm) with eluent A: water + 0.1% formic acid + 5 mM ammonium acetate and eluent B: methanol + 0.1% formic acid + 5 mM ammonium acetate. The flow rate was set to 0.5 ml per min. Elution starts with 95% eluent A, which linearly decreases to 75% over 0.5 min. This composition was held for 4 min before returning to initial conditions. The injected volume was 2 µl and the column temperature was set to 55 °C. 3-IAA and MOI were detected in MRM (multiple reaction monitoring) mode. The following transitions were monitored after positive electrospray ionization: 3-IAA: *m*/*z* 176.2 > 103.0; *m*/*z* 176.2 > 130.2; MOI: *m*/*z* 148.1 > 120.2; *m*/*z* 148.1 > 130.2; *m*/*z* 148.1 > 133.1. Quantification was done according to a standard curve.

### Isolation of immune cells and flow cytometry

Tumours were taken and cut into similar-sized pieces. Tumours were rinsed with cold PBS and digested in RPMI (Sigma, 61870044) supplemented with 10% FBS (Gibco, 10500064), 2.5 mg ml^−1^ collagenase D (Roche, 11088866001) and 0.2 mg ml^−1^ DNase l (Roche, 11284932001) for 35 min at 37 °C with continuous shaking. Afterwards, the suspension was strained through a 40-µm cell strainer and quenched with cold PBS. Subsequently, immune or tumour cells were stained with Fc block and live/dead staining (Thermo Fisher Scientific L34957 and L10119) for 30 min in the dark. Afterwards cells were washed, stained with the indicated flow cytometry antibodies and incubated for 30 min in the dark again. Flow cytometry was performed on a Fortessa flow cytometer (BD). To assess the cytokine profile of immune cells, restimulation of T cells with 50 ng ml^−1^ PMA, 500 ng ml^−1^ ionomycin and 1 µg ml^−1^ brefeldin A was performed for 3 h at 37 °C. After surface staining, cells were fixed and permeabilized using the eBioscience Foxp3 intracellular staining kit (00-5523-00). The following intracellular antibodies were used: IFNγ (1:200 dilution) and TNF (1:400 dilution). The following surface antibodies were used to classify lymphocytes (CD3 (1:300 dilution), CD4 (1:500 dilution), CD8 (1:400 dilution), CD19 (1:100 dilution), CD44 (1:500 dilution), PD-1 (1:400 dilution) and NK1.1 (1:400 dilution)) or myeloid cells (CD11b (1:800 dilution), CD11c (1:300 dilution), CD45 (1:800 dilution), Ly6G (1:800 dilution), Ly6C (1:600 dilution), Ly6B (1:200 dilution), CD115 (1:300 dilution), F4/80 (1:600 dilution) and MHCll (1:600 dilution)). Epithelial cancer cells were stained with EPCAM (1:200 dilution) antibody. For Ly6B staining, the goat anti-rat IgG antibody (1:400 dilution) was used. Cells were counted by flow cytometry using 2,500 to 5,000 beads (Spherotec, ACBP 100-10). Viability of neutrophils in vitro was assessed using PI/Annexin V staining according to the manufacturer’s protocol. ROS expression was determined in immune cells in vivo after tissue digestion as described above. Afterwards, the ROS dye CellROX (Thermo Fisher Scientific, C10422) was stained in parallel with flow cytometry antibodies at a dilution of 1:1,000. Software analysis and histogram generation was carried out using FlowJo v.10.

### Cell culture

KPC cells were obtained from Ximbio under catalogue number 153474; Hy19636_GLRM reporter cells and mSt-ATG4B/mSt cells were provided by A. Kimmelman^31^; MC38 and LLC-GFP (ATCC) cells were provided by A. Giannou; and MIA PaCa-2, BxPC-3 and T3M-4 cells (all ATCC) were provided by C. Güngör. All cells tested negative for mycoplasma contamination. Cells were maintained under standard conditions at 37 °C and 5% CO_2_ and regularly visually inspected. Cells were grown in DMEM GlutaMAX (Thermo Fisher Scientific, 10566016) supplemented with penicillin and streptomycin (Gibco, 15140122) and 10% FBS (Gibco, 10500064). For in vitro experiments, cancer cells were plated at 5,000–10,000 cells per well in a 96-well plate. Cells were allowed to seed overnight, except in experiments with neutrophil co-culture, treatment of GPX-knockdown cells or co-treatment with MPO. Subsequently, treatment with indicated compounds and treatment duration was initiated. 3-IAA or 3-IPA (3-IPA, Sigma, 57400; 3-IAA, Sigma, I3750) were dissolved in 1 M NaOH in PBS and PH-adjusted to 7.4 using 1 M HCl or DMSO when indicated. NAC was diluted in PBS and used at a concentration of 1 mM. H_2_O_2_ (Sigma, H1009) was used at a concentration of 400 µM.

Tumour cell viability and proliferation was assessed using a MTT or MTS assay (Abcam, ab201191 and ab197010) according to the manufacturer’s protocol. Absorbance was assessed using the FLUOstar Omega (BMG Labtech). In other experiments, viability was assessed by flow cytometry using SYTOX (Thermo Fisher Scientific, S34857), PI (Biolegend, 421301) and 2,500–5,000 counting beads (Spherotec, ACBP 100-10) as a reference. In parallel, intracellular ROS was measured using CellROX (Thermo Fisher Scientific, C10422) according to the manufacturer’s protocol and assessed by flow cytometry.

Neutrophils or T cells were isolated from bone marrow or spleens of healthy, untreated mice and sorted using fluorescence-activated cell sorting (FACS) as LY6G^+^CD11b^+^ and TCRβ^+^CD4^+^ or CD8^+^, respectively. Cells were seeded at a concentration of 20,000–50,000 per well in a 96-well plate. Viability of neutrophils or T cells was assessed by flow cytometry using SYTOX (Thermo Fisher Scientific, S34857) or PI/Annexin V staining (Biolegend, 640914) at the indicated time of the experiment. In some experiments, 200 mU ml^−1^ MPO or 400 mU ml^−1^ MPO (Merck, 475911) or indicated concentrations of MOI (Sigma, 493397) were added. Degranulation of neutrophils was assessed by the release of MPO. A total of 1 × 10^6^ neutrophils were sorted using FACS as described above. Neutrophils were incubated in HBSS (Gibco, 14065-56) and the indicated treatment, or *N*-formylmethionyl-leucyl-phenylalanine (fMLP; Merck, F3506), as a positive control, was added. After 30 min of incubation, MPO activity was measured using an MPO activity assay kit (Abcam, ab105136) according to the manufacturer’s protocol. NET formation of neutrophils was determined using SYTOX stain after 3 h of incubation with the indicated compounds or 100 nM phorbol-12-myristate-13-acetate (PMA; Merck, 524400) as a positive control. NETs were measured as SYTOX-positive cells using flow cytometry.

Pre-neutrophils were sorted by FACS (lineage-negative (CD3,NK1.1,CD19,B220)^−^, CD115^−^, Ly6B^+^, Ly6G^int-low^) as previously described^26^ and cultured at a density of 20,000–50,000 per well in a 96-well plate. Indicated treatments were applied and viability was assessed using PI staining in flow cytometry.

### MPO activity

Intratumoural or bone-marrow-derived neutrophils (LY6G^+^CD11b^+^) or pre-neutrophils (lineage-negative (CD3,NK1.1,CD19,B220)^−^, CD115^−^, Ly6B^+^, Ly6G^int-low^) were sorted by FACS. A total of 50,000 cells were processed for MPO activity measurement using an MPO activity assay kit according to the manufacturer’s protocol (Abcam, ab219925). Fluorescence was analysed using FLUOstar Omega (BMG Labtech). MPO activity was calculated based on a standard curve as suggested by the manufacturer’s protocol.

### Protein screen

Tumour tissue from three individual mice per group was pooled and proteins were extracted using scioExtract buffer (Sciomics). The samples were labelled for 2 h with scioDye 2 (Sciomics) at an adjusted protein concentration. The reference sample was labelled with scioDye 1 (Sciomics). After 2 h, the reaction was stopped and the buffer was exchanged with PBS. All labelled protein samples were stored at −20 °C until use. The samples were analysed in a dual-colour approach using a reference-based design on scioDiscover antibody microarrays (Sciomics). The arrays were blocked with scioBlock (Sciomics) on a Hybstation 4800 (Tecan) and afterwards the samples were incubated competitively with the reference sample using a dual-colour approach. After incubation for 3 h, the slides were thoroughly washed with 1× PBSTT, rinsed with 0.1× PBS as well as with water and subsequently dried with nitrogen. Slide scanning was conducted using a Powerscanner (Tecan) with constant instrument laser power and PMT settings. Spot segmentation was performed with GenePix Pro 6.0 (Molecular Devices). Acquired raw data were analysed using the (LIMMA) package of R-Bioconductor after uploading the median signal intensities. For normalization, a specialized invariant Lowess method was applied. Downregulated proteins (M-value of < −0.35) or upregulated proteins (>0.35) in the 3-IAA and FIRINOX sample were uploaded to the STING database (https://string-db.org/cgi/input.pl). The standard pipeline was used to perform KEGG enrichment analysis. Only pathways with a false discovery rate (FDR) < 0.05 were considered statistically significant.

### RNA extraction and mRNA sequencing

Fresh frozen tumour tissue was lysed in TRIzol reagent (Thermo Fisher Scientific, 15596018) and RNA was extracted using the chloroform-isopropanol method. mRNA was purified from total RNA using poly-T oligo-attached magnetic beads. After fragmentation, the first-strand cDNA was synthesized using random hexamer primers followed by second-strand cDNA synthesis. The library was ready after end repair, A-tailing, adapter ligation, size selection, amplification and purification. The library was checked with Qubit and qPCR for quantification, and Bioanalyzer for size distribution detection. Quantified libraries were sequenced on the Illumina platform with at least 60 million reads per sample.

Sequence reads were processed with fastp (v0.20.1) to remove sequences of sequencing adapters and low-quality (Phred quality score below 15) sequences from the 3′ end of the sequence reads. Afterwards, reads were aligned to the mouse reference assembly (GRCm39.104) using STAR (v.2.7.9a)^47^. Differential expression was assessed with DESeq2 (ref. ^48^). A gene was considered significantly differentially expressed if the corresponding absolute log_2_-transformed fold change (log2FC) was no less than 0.6 and, in addition, the FDR did not exceed a value of 0.1. The gene set enrichment analysis (GSEA) of the Reactome pathway Autophagy (R-HSA-9612973) was performed using fgsea (v.4.1)^49^.

### qPCR

Total RNA was extracted from cell lines using Trizol Reagent (Invitrogen,15596018) and the total RNA extraction kit (Qiagen, 74004/74104) according to the manufacturer’s protocol. The High-Capacity cDNA Synthesis Kit (Thermo Fisher Scientific, 4368813) was used for cDNA synthesis. Primers and probes were purchased from Applied Biosystems. Mouse primers and probes are: *Gpx3* (Mm00492427_m1), *Gpx7* (Mm00481133), *Ahr* (Mm00478932_m1). qPCR was performed using the TaqMan Master Mix (Thermo Fisher Scientific, 4369016) on the StepOne Plus system (Applied Biosystems). Forty to 44 cycles were applied for every assay and technical doublets or triplicates were used. If at least two out of three values of the technical replicates were undetectable, the expression was considered non-detectable. Relative expression was normalized to *Gapdh* (Mm99999915_g1).

### Lentiviral transfer of shRNAs

Lentiviral vectors expressing short hairpin RNAs (shRNAs) under control of the human U6 promoter (MISSION pLKO.1-puro) directed against mouse *Gpx3* (TRCN000076539), mouse *Gpx7* (TRCN0000076563) and mouse *Ahr* (TRCN0000218025) as well as a nontargeted control shRNA (SHC002, scrambled) were obtained from Sigma-Aldrich. The production of lentiviral particles has been described in detail elsewhere^50^, and protocols are available online (http://www.LentiGO-Vectors.de). For the transduction of KPC cells with the HIV-1-derived lentiviral vectors, 2.5 × 10^4^ cells were plated in 0.5 ml medium with 8 μg ml^−1^ polybrene per well of a 24-well plate. After plating, the addition of 10 μl VSV-G pseudotyped, non-concentrated lentiviral particles led to the stable integration of shRNAs and the puromycin resistance gene into the cell’s genome. To increase the transduction rate by spin-inoculation, the plate was centrifuged at 1,000*g* and 25 °C for 1 h. The selection of successfully transduced cells with 1 μg ml^−1^ puromycin in the culture medium was started four days after transduction.

### Immunohistochemistry and quantification

Tissues were fixed in 4% formalin in PBS and embedded in paraffin using an ASP300S dehydration machine (Leica) and an EG1160 tissue embedding system (Leica). Paraffin sections (2 µm) were cut and stained with H&E or processed for immunohistochemistry as follows: after dewaxing and inactivation of endogenous peroxidases (3% hydrogen peroxide in PBS), antibody-specific heat-mediated antigen retrieval was performed using the Ventana Benchmark XT machine (Ventana). Sections were blocked (10% FCS in PBS) and then incubated with anti-LC3B antibody (1:400 dilution, Thermo Fisher Scientific, PA1-46286); anti-nitrotyrosine antibody (1:100 dilution, Thermo Fisher Scientific, A-21285); anti-CC3 antibody (1:100 dilution, Cell Signaling, 9661); p62/SQSTM1 (1:500 dilution, Thermo Fisher Scientific, PA5-20839); and anti-Ki67 antibody (1:100 dilution, Abcam, 15580). For detection of specific binding, the Ultra View Universal DAB Detection Kit (Ventana, Roche) was used, which contains secondary antibodies, DAB stain and haematoxylin counterstaining reagent. Slides were scanned using NanoZoomer 2.0-HT (Hamamatsu Photonics) and representative images were taken using Fiji.

Quantification for LC3B, Ki67, nitrotyrosine and CC3 was done in a blinded manner. Positive cells were determined using ImageJ v.2.1.0/1.53c and the threshold was adjusted according to the staining intensity of the respective antibody and maintained for all tumours analysed with the same staining. The number of positive cells was counted per 250–500 × 500-μm field (10× to 40× magnification) in three to five fields per sample.

### Widefield microscopy

To visualize the GFP-LC3-RFP reporter signal expression of Hy19636_GLRM PDAC cells, tissues were fixed in 2% PFA solution at 4 °C overnight, incubated in PBS containing 30% sucrose and embedded in Tissue-Tek OCT compound (Sakura Finetek) on dry ice. For further analysis, 7-μm sections were used. Widefield imaging was performed using the THUNDER Imager 3D Live Cell and 3D Cell Culture (Leica Microsystems) equipped with a 40× 1.10 NA water immersion objective. LED power and exposure time and other system-specific settings were first optimized using positive control tissue and not changed between image acquisitions of the different groups to provide optimal comparability. For each tissue section, an average of five regions of interest were randomly selected and imaged for subsequent quantitative analysis. ImageJ imaging software was used for file navigation, adjustment of colour balance and image analysis. GFP and RFP quantification was determined using ImageJ v.2.1.0/1.53c. The mean fluorescence intensity for each respective signal was determined per slide in at least five areas per tumour.

### Graphical abstracts

Figure 1a, Extended Data Fig. 2a,d and small icons in Figs. 1–4 were created with BioRender.com.

### Statistical analyses

All statistical analyses, unless otherwise indicated, were performed using GraphPad Prism 9.3.1. Normality and log-normality were tested using Shapiro–Wilk or Kolmogorov–Smirnov tests. If normality was not given, non-parametric testing was performed. Tests were performed two-sided if not otherwise indicated and resulting significant (*P* < 0.05) *P* values are shown.

### Reporting summary

Further information on research design is available in the Nature Portfolio Reporting Summary linked to this article.

## Online content

Any methods, additional references, Nature Portfolio reporting summaries, source data, extended data, supplementary information, acknowledgements, peer review information; details of author contributions and competing interests; and statements of data and code availability are available at 10.1038/s41586-023-05728-y.

## Supplementary information

Reporting Summary

Supplementary Table 1 Protein screen comparing tumours isolated from 3-IAA and FIRINOX-treated mice to those isolated from FIRINOX-only-treated mice (control). Protein screen was performed and analysed as described in the Methods.

Supplementary Table 2 KEGG pathways of proteins downregulated in tumours isolated from 3-IAA and FIRINOX-treated mice. Proteins with a difference of < −0.35 compared to FIRINOX-only-treated tumours (Table 1) were selected and analysed as described in the Methods.

Supplementary Table 3 KEGG pathways of proteins upregulated in tumours isolated from 3-IAA and FIRINOX-treated mice. Proteins with a difference of > +0.35 compared to FIRINOX-only-treated tumours (Table 1) were selected and analysed as described in the Methods.

## Source data

Source Data Fig. 1

Source Data Fig. 2

Source Data Fig. 3

Source Data Fig. 4

Source Data Extended Data Fig. 1

Source Data Extended Data Fig. 2

Source Data Extended Data Fig. 3

Source Data Extended Data Fig. 4

Source Data Extended Data Fig. 5

Source Data Extended Data Fig. 6

Source Data Extended Data Fig. 7

Source Data Extended Data Fig. 8

Source Data Extended Data Fig. 9

## Figures and Tables

**Extended Data Table 1:**
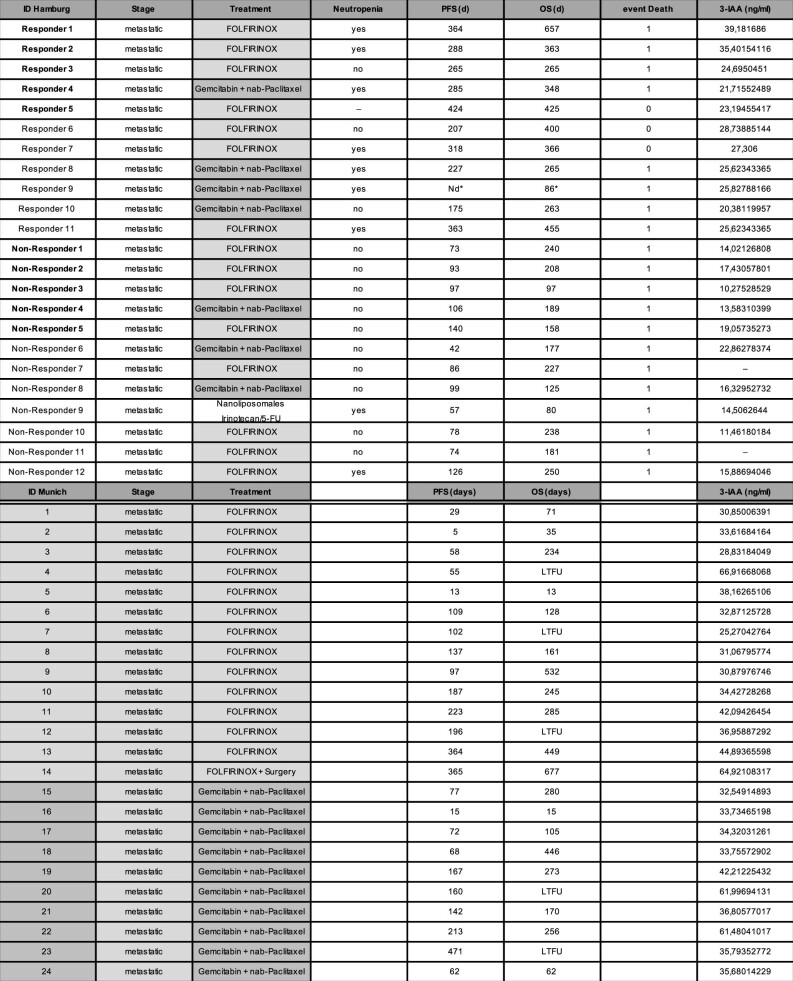
Patient data for the Hamburg (observation) and Munich (validation) cohorts

3-IAA was measured by CLIA as described in the Methods. Response was defined by tumour regression of at least 25% in CT scan or at least unchanged tumour size with either >40% drop in serum tumour marker and/or PFS > 140 days (Hamburg cohort only). Patients whose tumours progressed within the first 140 days of treatment were considered non-responders (Hamburg cohort only). Neutropenia was defined as a neutrophil count of less than 1.5 × 10^9^ per litre as measured by the hospital routine standard at its lowest point during the first three months of chemotherapy. 3-IAA was measured using CLIA after two to three cycles of chemotherapy (Hamburg cohort) or before chemotherapeutic treatment (Munich cohort). LTFU are patients who were lost to follow-up. Patient marked with (*) died of a cause other than progressive disease.

## Data Availability

RNA-seq data have been submitted to the European Nucleotide Archive (ENA). They are publicly available under accession number PRJEB58222. Shotgun metagenomic sequencing data were filtered for human reads and are available under accession number PRJEB58222. Source data are provided with this paper.
