# Author Correction: Microbiota-derived 3-IAA influences chemotherapy efficacy in pancreatic cancer

**DOI:** 10.1038/s41586-025-08979-z

**Published:** 2025-05-13

**Authors:** Joseph Tintelnot, Yang Xu, Till R. Lesker, Martin Schönlein, Leonie Konczalla, Anastasios D. Giannou, Penelope Pelczar, Dominik Kylies, Victor G. Puelles, Agata A. Bielecka, Manuela Peschka, Filippo Cortesi, Kristoffer Riecken, Maximilian Jung, Lena Amend, Tobias S. Bröring, Marija Trajkovic-Arsic, Jens T. Siveke, Thomas Renné, Danmei Zhang, Stefan Boeck, Till Strowig, Faik G. Uzunoglu, Cenap Güngör, Alexander Stein, Jakob R. Izbicki, Carsten Bokemeyer, Marianne Sinn, Alec C. Kimmelman, Samuel Huber, Nicola Gagliani

**Affiliations:** 1 II. Department of Medicine, University Medical Center Hamburg-Eppendorf, Hamburg, Germany; 2 Mildred Scheel Cancer Career Center HaTriCS4, University Medical Center Hamburg-Eppendorf, Hamburg, Germany; 3 Department of General, Visceral and Thoracic Surgery, University Medical Center Hamburg-Eppendorf, Hamburg, Germany; 4 Research Group Microbial Immune Regulation, Helmholtz Centre for Infection Research, Braunschweig, Germany; 5 I. Department of Medicine, University Medical Center Hamburg- Eppendorf, Hamburg, Germany; 6 Hamburg Center for Translational Immunology (HCTI), Hamburg, Germany; 7 III. Department of Medicine, University Medical Center Hamburg-Eppendorf, Hamburg, Germany; 8 Department of Clinical Medicine, Aarhus University, Aarhus, Denmark; 9 Institute of Clinical Chemistry and Laboratory Medicine, University Medical Center Hamburg-Eppendorf, Hamburg, Germany; 10 Newborn Screening and Metabolic Laboratory, Department of Pediatrics, University Medical Center Hamburg-Eppendorf, Hamburg, Germany; 11 Research Department Cell and Gene Therapy, Department of Stem Cell Transplantation, University Medical Center Hamburg-Eppendorf, Hamburg, Germany; 12 Bridge Institute of Experimental Tumor Therapy, West German Cancer Center, University Hospital Essen, University Duisburg-Essen, Essen, Germany; 13 Division of Solid Tumor Translational Oncology, German Cancer Consortium (DKTK Partner Site Essen) and German Cancer Research Center (DKFZ), Heidelberg, Germany; 14 Irish Centre for Vascular Biology, School of Pharmacy and Biomolecular Sciences, Royal College of Surgeons in Ireland, Dublin, Ireland; 15 Center for Thrombosis and Hemostasis (CTH), Johannes Gutenberg University Medical Center, Mainz, Germany; 16 Department of Internal Medicine III, Ludwig-Maximilians-University (LMU) Hospital, Munich, Germany; 17 Hannover Medical School (MHH), Hannover, Germany; 18 Hematology–Oncology Practice Hamburg (HOPE), University Cancer Center Hamburg, Hamburg, Germany; 19 Department of Radiation Oncology, Perlmutter Cancer Center, New York University Grossman School of Medicine, New York, NY USA

**Keywords:** Cancer therapeutic resistance, Tumour heterogeneity, Predictive markers, Chemotherapy

Correction to: *Nature* 10.1038/s41586-023-05728-y Published online 22 February 2023

We found that a data processing error occurred during the visualization of metabolite concentrations in Fig. 1d and 1e of our article. More specifically, the concentration values of metabolites below 100 ng/ml are usually exported with decimals and indicated with a period, but the periods were automatically converted to commas and no longer recognized as decimal indicators by the software while generating Fig. 1d and 1e.

**Fig. 1:**
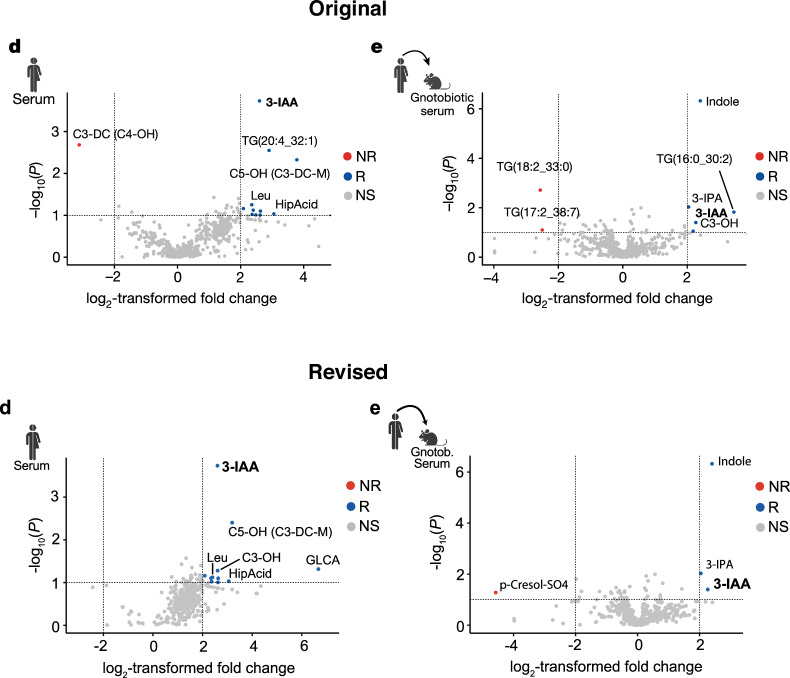
Original and revised Fig. 1d,e .

The original dataset of all metabolite concentrations was not affected, nor was the visualization of the key metabolite 3-IAA or the control metabolites HipAcid and 3-IPA. Consequently, none of the conclusions of our study are affected.

The formatting error affected the visualization of some of the metabolites in the non-specified (NS) background area of the volcano plots (dots colored in grey) and of seven metabolites that were initially annotated in the figures (i.e., d: C3-DC (C4-OH), TG(20:4_32:1), C5-OH (C3-DC-M); e: TG(18:2_33:0), TG(17:2_38:7), TG(16:0_30:2), C3-OH). These metabolites were irrelevant to our conclusions and thus not mentioned in the text.

For completeness and clarity, in the corrected version, we have labeled three additional metabolites that are increased in the serum of R patients, i.e., GLCA, C3-OH (see Fig. 1: revised Fig. 1d, below) or NR-microbiota colonized mice, i.e., p-Cresol-SO4 (see Fig. 1: revised Fig. 1e, below) and removed any metabolite indicators. Source data for Fig. 1 have now been revised online.

In addition, in Extended Data Fig. 8c, we have now applied only one post-hoc test for consistency. Applying the Dunnett’s post hoc test changes the *p*-values from “0.028” to “0.022” and from “0.026” to “0.047.”

We detected and corrected typographical errors in Fig. 2b (from “0.0285” to “0.0236”), Fig. 4g (from “<” to “=”), in the legend of Extended Data Fig. 2i (from “0901”/“i2” to “0903”), and in Extended Data Fig. 4a (from “IFN-γ” / “TNF-α” to “TNF-α BV421” on the dot plot *x* axis). The changes are reflected in the HTML and PDF versions of the article. In Extended Data Table 1, the comma should be considered a decimal indicator.

