# Supplementary material for: Microbiota-derived 3-IAA influences chemotherapy efficacy in pancreatic cancer
Source: Nature. 2023 Feb 22;615(7950):168–74. doi: 10.1038/s41586-023-05728-y (PMC9977685; doi:10.1038/s41586-023-05728-y)
Supplement: Supplementary file 1 — Reporting Summary [file 41586_2023_5728_MOESM1_ESM.pdf]

nature portfolio

Corresponding author(s): Joseph Tintelnot, Nicola Gagliani

Last updated by author(s): Dec 14, 2022

Reporting Summary

Nature Portfolio wishes to improve the reproducibility of the work that we publish. This form provides structure for consistency and transparency in reporting. For further information on Nature Portfolio policies, see our Editorial Policies and the Editorial Policy Checklist.

Statistics

For all statistical analyses, confirm that the following items are present in the figure legend, table legend, main text, or Methods section.

n/a	Confirmed	
☐	☒	The exact sample size (*n*) for each experimental group/condition, given as a discrete number and unit of measurement
☐	☒	A statement on whether measurements were taken from distinct samples or whether the same sample was measured repeatedly
☐	☒	The statistical test(s) used AND whether they are one- or two-sided<br>*Only common tests should be described solely by name; describe more complex techniques in the Methods section.*
☐	☒	A description of all covariates tested
☐	☒	A description of any assumptions or corrections, such as tests of normality and adjustment for multiple comparisons
☐	☒	A full description of the statistical parameters including central tendency (e.g. means) or other basic estimates (e.g. regression coefficient) AND variation (e.g. standard deviation) or associated estimates of uncertainty (e.g. confidence intervals)
☐	☒	For null hypothesis testing, the test statistic (e.g. *F*, *t*, *r*) with confidence intervals, effect sizes, degrees of freedom and *P* value noted<br>*Give P values as exact values whenever suitable.*
☒	☐	For Bayesian analysis, information on the choice of priors and Markov chain Monte Carlo settings
☒	☐	For hierarchical and complex designs, identification of the appropriate level for tests and full reporting of outcomes
☐	☒	Estimates of effect sizes (e.g. Cohen's *d*, Pearson's *r*), indicating how they were calculated

Our web collection on statistics for biologists contains articles on many of the points above.

Software and code

Policy information about availability of computer code

Data collection	Flow cytometry data were collected using FACSDiva version 8 on a Fortessa flow cytometer; widefield images were obtained using Leica Application Suite X software v.3.6.0; MTT and CLIA were analyzed using FLUOstar Omega; Protein screen was acquired using GenePix Pro 6.0., Illumina MiSeq v3 was used for 16S rRNA sequencing and Illumina NovaSeq for shotgun metagenomic sequencing; Xevo TQ-S mass spectrometer was used for metabolomic screening; StepOne Plus system for rt-PCR and NanoZoomer 2.0-HT for slide scanning
Data analysis	The following software were used for data analysis: MetaboAnalyst v. 5.0; ImageJ v. 2.1.0/1.53c; Prism 9.3.1; R version 4.1; FlowJo v10.4; BBMap version 36.49; SPAdes 3.1.5.2; MetaWrap 1.2; fastp v0.20.1; STAR v2.7.9a; fgsea v4.1; DADA2; DESeq2; LIMMA; phyloseq; LEfSe

For manuscripts utilizing custom algorithms or software that are central to the research but not yet described in published literature, software must be made available to editors and reviewers. We strongly encourage code deposition in a community repository (e.g. GitHub). See the Nature Portfolio guidelines for submitting code & software for further information.

Data

Policy information about availability of data

All manuscripts must include a data availability statement. This statement should provide the following information, where applicable:

- Accession codes, unique identifiers, or web links for publicly available datasets
- A description of any restrictions on data availability
- For clinical datasets or third party data, please ensure that the statement adheres to our policy

RNA-seq data have been submitted to the European Nucleotide Archive (ENA). They are publicly available under accession number PRJEB58222. Filtered human shotgun metagenomic sequencing data of stool samples from the Hamburg cohort are available under accession number PRJEB58222. Source data are provided for all figures. Protein screening data is provided in Supplementary Information table 1-3.

Field-specific reporting

Please select the one below that is the best fit for your research. If you are not sure, read the appropriate sections before making your selection.

☒ Life sciences ☐ Behavioural & social sciences ☐ Ecological, evolutionary & environmental sciences

For a reference copy of the document with all sections, see nature.com/documents/nr-reporting-summary-flat.pdf

Life sciences study design

All studies must disclose on these points even when the disclosure is negative.

Sample size	Sample sizes were based on experience with the described models as well as small pilot experiments with up to 5 mice per group for new models. Regarding human data, no sample size was calculated; rather metabolomic screening was performed on a small pilot group. In the following time of recruitment, all eligible patients were allowed to enroll into the study.
Data exclusions	No data or animals were excluded from analysis, except for clear technical failure.
Replication	Experiments were repeated multiple times with similar results as indicated in the figure legends. All critical in vivo experiments were reproduced using the same system (e.g. Chemotherapy treatment in R colonized mice using the same donor and mice) and/ or using orthogonal approaches (e.g. endogenous 3-IAA production by R microbiota or 3-IAA gavage in SPF or NR microbiota colonized mice).
Randomization	Prior to treatment initiation mice bearing tumors were randomized. Tumor sizes were not measured at the time of randomization.<br>Human study participants were not randomized, because the caring physician decided about the treatment and the individual response to therapy defined the allocation to either R or NR group.
Blinding	Blinding was not performed in mouse experiments. The investigator needed to know the treatment groups in order to perform the study. Tumor weights (an objective measurement) and tumor sizes were carried out only at the study endpoints after mice were euthanized and tumors were harvested, if possible together with a blinded rater.<br>No active blinding was performed for human materials since patients were treated as defined by the caring physician and the investigator needed to know the response of the specific patient in order to define the allocation to the R or NR group. Still, at the time of stool and serum sample collection, the response of the individual patient was not defined.

Reporting for specific materials, systems and methods

We require information from authors about some types of materials, experimental systems and methods used in many studies. Here, indicate whether each material, system or method listed is relevant to your study. If you are not sure if a list item applies to your research, read the appropriate section before selecting a response.

Materials & experimental systems

n/a	Involved in the study	
☐	☒	Antibodies
☐	☒	Eukaryotic cell lines
☒	☐	Palaeontology and archaeology
☐	☒	Animals and other organisms
☐	☒	Human research participants
☒	☐	Clinical data
☒	☐	Dual use research of concern

Methods

n/a	Involved in the study	
☒	☐	ChIP-seq
☐	☒	Flow cytometry
☒	☐	MRI-based neuroimaging

Antibodies

Antibodies used

The following antibodies were used for intracellular flow cytometry: IFN-γ-BUV737 (BD Horizon, 612769, XMG1.2, 0276632), TNF-α-BV421 (Biolegend, 506327, MP6-XT22, B293819)

The following surface antibodies were used for flow cytometry: CD3-BUV395 (BD Horizon, 563565, 145-2C11, 1117788), CD4-BUV737 (BD Horizon, 612844, RM4-5, 1056992), CD8-BV650 (Biolegend, 301041, RPA-T8), PD-1-BV711 (Biolegend, 135231, 29F.1A12, B317246), CD11b-BV510 (BD Horizon, 562950, M1/70, 5306509), CD11c-PE (BD Bioscience, 553802, HL3, 6301690), CD45-BV785 (Biolegend, 103149, 30-F11, B336128), CD45-APC/Cy7 (Biolegend, 103115, 30-F11, B291572), Ly6G-Alexa647 (Biolegend, 127609, 1A8, B255839), Ly6C-BV570 (Biolegend, 128029, HK1.4, B310125), F4/80-BV421 (Biolegend, 123137, BM8, B242665), MHCll-Alexa700 (Biolegend, 107621, M5/114.15.2), CD19-APC/Cy7 (Biolegend, 115530, 6D5), CD19-BV421 (Biolegend, 115549, 6D5), CD115-BV421 (Biolegend, 135513, AFS98), Ly6B (Abcam, ab53457, 7/4), NK1.1-BV421 (Biolegend, 108741, PK136), EPCAM-Alexa488 (Biolegend, 118210, G8.8), CD3-BV421 (Biolegend, 100227, 17A2).
The following secondary antibody was used: Goat anti-Rat IgG (Thermo Fischer, A-11006, polyclonal).

The following antibodies were used for IHC: anti-LC3B antibody (Thermo Fischer Scientific, PA1-46286), anti-Nitrotyrosine antibody

(Thermo Fischer Scientific, A-21285), anti-CC3 antibody (Cell Signaling, 9661), anti p62/PQSTM1 (Thermo Fischer, PA5-20839) and anti-Ki67 antibody (Abcam, 15580).

Counter stain for IHC was done using the Universal DAB Detection Kit (Ventana, Roche, 760-500)

In vivo depletion of immune cells was achieved using: anti-CD8 antibody clone 53-6.7 (BioXcell, BE0004), anti-CD4 antibody clone GK1.5 (BioXcell, BE0003), isotype control Clone 2A3 (BioXcell, BE0089).

Validation

IFN-γ-BUV737 (BD Horizon, https://www.bdbiosciences.com/en-au/products/reagents/flow-cytometry-reagents/research-reagents/single-color-antibodies-ruo/buv737-rat-anti-mouse-ifn.612769)
TNF-α-BV421 (Biolegend, https://www.biolegend.com/en-us/sean-tuckers-tests/brilliant-violet-421-anti-mouse-tnf-alpha-antibody-7336)
CD3-BUV395 (BD Horizon, https://www.bdbiosciences.com/en-us/products/reagents/flow-cytometry-reagents/research-reagents/single-color-antibodies-ruo/buv395-hamster-anti-mouse-cd3e.563565)
CD4-BUV737 (BD Horizon, https://www.bdbiosciences.com/en-au/products/reagents/flow-cytometry-reagents/research-reagents/single-color-antibodies-ruo/buv737-rat-anti-mouse-cd4.612844)
CD8-BV650 (Biolegend, https://www.biolegend.com/en-us/products/brilliant-violet-650-anti-human-cd8a-antibody-7652?GroupID=BLG5903)
PD-1-BV711 (Biolegend, https://www.biolegend.com/nl-nl/products/brilliant-violet-711-anti-mouse-cd279-pd-1-antibody-12303)
CD11b-BV510 (BD Horizon, 562950, https://www.bdbiosciences.com/en-ch/products/reagents/flow-cytometry-reagents/research-reagents/single-color-antibodies-ruo/bv510-rat-anti-cd11b.562950)
CD11c-PE (BD Bioscience, 553802, https://www.bdbiosciences.com/en-au/products/reagents/flow-cytometry-reagents/research-reagents/single-color-antibodies-ruo/pe-hamster-anti-mouse-cd11c.553802)
CD3-BV421 (Biolegend, https://www.biolegend.com/fr-ch/products/brilliant-violet-421-anti-mouse-cd3-antibody-7326)
EPCAM-Alexa488 (Biolegend, https://www.biolegend.com/fr-ch/products/alexa-fluor-488-anti-mouse-cd326-ep-cam-antibody-4972)
NK1.1-BV421 (Biolegend, https://www.biolegend.com/fr-ch/products/brilliant-violet-421-anti-mouse-nk-1-1-antibody-7150)
CD115-BV421 (Biolegend, https://www.biolegend.com/fr-ch/products/brilliant-violet-421-anti-mouse-cd115-csf-1r-antibody-8971)
CD19-BV421 (Biolegend, https://www.biolegend.com/fr-ch/products/brilliant-violet-421-anti-mouse-cd19-antibody-7160)
CD19-APC/Cy7 (Biolegend, https://www.biolegend.com/fr-ch/products/apc-cyanine7-anti-mouse-cd19-antibody-3903)
CD45-BV785 (Biolegend, https://www.biolegend.com/en-us/products/brilliant-violet-785-anti-mouse-cd45-antibody-10636?GroupID=BLG1932)
CD45-APC/Cy7 (Biolegend, https://www.biolegend.com/en-us/products/apc-cyanine7-anti-mouse-cd45-antibody-2530?GroupID=BLG1932)
Ly6G-Alexa647 (Biolegend, https://www.biolegend.com/en-us/products/alexa-fluor-647-anti-mouse-ly-6g-antibody-4780)
Ly6C-BV605 (Biolegend, https://www.biolegend.com/en-us/products/brilliant-violet-570-anti-mouse-ly-6c-antibody-7392?GroupID=BLG5853)
F4/80-BV421 (Biolegend, https://www.biolegend.com/en-us/products/brilliant-violet-421-anti-mouse-f4-80-antibody-7199?GroupID=BLG5319)
MHCll-Alexa700 (Biolegend, https://www.biolegend.com/en-us/products/alexa-fluor-700-anti-mouse-i-a-i-e-antibody-3413?GroupID=BLG4736)
Ly6B (Abcam, https://www.abcam.com/neutrophil-antibody-74-ab53457.html)
SQSTM1 (Thermo Fischer, https://www.thermofisher.com/antibody/product/SQSTM1-Antibody-Polyclonal/PA5-20839)
anti-LC3B antibody (Thermo Fischer Scientific, https://www.thermofisher.com/antibody/product/LC3B-Antibody-Polyclonal/PA1-46286);
anti-Nitrotyrosine antibody (Thermo Fischer Scientific, https://www.thermofisher.com/antibody/product/Nitrotyrosine-Antibody-Polyclonal/A-21285);
anti-CC3 antibody (Cell Signaling, https://www.cellsignal.com/products/primary-antibodies/cleaved-caspase-3-asp175-antibody/9661)
anti-Ki67 antibody (Abcam, https://www.abcam.com/ki67-antibody-ab15580.html)
anti-CD8 antibody (BioXcell,https://bxcell.com/product/m-cd8a/)
anti-CD4 antibody (BioXcell, https://bxcell.com/product/m-cd4/)
isotype control (BioXcell, https://bxcell.com/product/rat-igg2a-isotype-control/)
Universal DAB Kit (https://www.bfarm.de/SharedDocs/Kundeninfos/DE/08/2011/02264-11_kundeninfo_de.pdf__blob=publicationFile&v=4)
anti-Rat IgG antibody (Thermo Fischer, https://www.thermofisher.com/antibody/product/Goat-anti-Rat-IgG-H-L-Cross-Adsorbed-Secondary-Antibody-Polyclonal/A-11006)

Eukaryotic cell lines

Policy information about cell lines

Cell line source(s)

KPC cells were obtained from Ximbio under catalogue number 153474; Hy19636_GLRM reporter cells, mSt-ATG4B and mSt were produced and provided by Alec Kimmelman (Yang et al. Cancer discovery 2018); MIA PaCa-2, BxPC-3 and T3M-4 (all from ATCC); MC38 and LLC-GFP (ATCC) were provided by Anastasios Giannou.

Authentication

The murine cell line KPC acquired from Ximbio was visually inspected and carefully maintained in a central lab cell bank. All other cells were maintained in a central lab cell bank and regularly visually inspected for changes.

Mycoplasma contamination

Routine testing for mycoplasma was conducted by PCR. All cell lines tested negative for mycoplasma.

Commonly misidentified lines
(See ICLAC register)

No commonly misidentified cell lines were used.

Animals and other organisms

Policy information about studies involving animals; ARRIVE guidelines recommended for reporting animal research

Laboratory animals	All mice used in this study were of a C57/BL6 background. Mice were kept under specific-pathogen-free or germ-free conditions, ambient temperature of 20±2°C, humidity of 55±10% and a dark/light cycle of 12 hours. Age- and sex-matched littermates between 4 and 16 weeks were mainly used. Both female and male mice were used, but within each experiment mice were sex-matched. MPO–/– bone marrow used to establish bone marrow chimeras was kindly provided by Prof. Baldus and Dr. Mollenhauer from University Hospital Cologne. AhR–/– bone marrow was provided by Prof Charlotte Esser.
Wild animals	This study did not involve wild animals.
Field-collected samples	This study did not involve samples collected from the field.
Ethics oversight	All mice were used in accordance with the institutional review board 'Behörde für Soziales, Familie, Gesundheit und Verbraucherschutz' (Hamburg, Germany).

Note that full information on the approval of the study protocol must also be provided in the manuscript.

Human research participants

Policy information about studies involving human research participants

Population characteristics	Patients diagnosed with mPDAC and scheduled for a treatment with GnP or FOLFIRINOX were recruited to the study (n=30). All patients received the chemotherapeutic treatment as their first treatment for metastatic disease. After exclusion of antibiotic treated patients or patients not receiving the chemotherapy, 23 patients remained on study. The median age in the responder group was 72 years (range: 47 to 82) and 65 years (range: 43 to 79) in the non-responder group. The sex distribution was 6 female and 5 male patients in the responder group and 6 female and 6 male patients in the non-responder group. All patients were suffering from metastatic disease and therefore both groups were strongly balanced for covariates.
Recruitment	All patients diagnosed with mPDAC and scheduled for a treatment with GnP or FOLFIRINOX at the participating centers between January 2020 and July 2021 were offered the chance to participate in this study (Hamburg cohort). Patients receiving antibiotics before start of treatment or during the first two months of treatment were excluded. Patients from the Munich cohort were retrospectively selected based on the received treatment and duration of response to guarantee a balanced representation of responder and non-responder patients.
Ethics oversight	Informed consent was obtained from all patients as approved by the ethics commission Hamburg (Ethikkommission der Ärztekammer Hamburg, Germany) or Informed consent was obtained from all patients as approved by the ethics commission Munich.

Note that full information on the approval of the study protocol must also be provided in the manuscript.

Flow Cytometry

Plots

Confirm that:

☒ The axis labels state the marker and fluorochrome used (e.g. CD4-FITC).

☒ The axis scales are clearly visible. Include numbers along axes only for bottom left plot of group (a 'group' is an analysis of identical markers).

☒ All plots are contour plots with outliers or pseudocolor plots.

☒ A numerical value for number of cells or percentage (with statistics) is provided.

Methodology

Sample preparation	Tumors were taken and cut into similar-sized pieces. Tumors were rinsed with cold PBS and digested in RPMI (Sigma, 61870044) supplemented with 10% FBS (Gibco, 10500064), 2,5mg/ml collagenase D (Roche, 11088866001) and 0.2mg/ml DNase I (Roche, 11284932001) for 35 minutes at 37°C with continuous shaking. Afterwards, the suspension was strained through a 40µm cell-strainer and quenched with cold PBS. Subsequently, immune or tumor cells were stained with Fc block and live/dead staining (Thermo Fischer Scientific L34957; L10119) for 30 minutes in the dark. Afterwards cells were washed, stained with indicated flow cytometry antibodies and again incubated for 30 minutes in the dark. Flow cytometry was performed on a Fortessa flow cytometer (BD). To assess the cytokine profile of immune cells, restimulation of T cells with 50ng/ml PMA, 500ng/ml ionomycin and 1µg/m brefeldin A was performed for 3 hours at 37°C. After surface staining, cells were fixed and permeabilized using the eBioscience Foxp3 intracellular staining kit (00-5523-00). The following intracellular antibodies were used: IFN-γ, TNF-α. The following surface antibodies to classify lymphocytes: i.e. CD3, CD4, CD8, CD19, PD-1, NK1.1 or myeloid cells: i.e. CD11b, CD11c, CD45, Ly6G, Ly6C, CD115, Ly6B, F4/80, MHCll were used. Software analysis and histogram generation was carried out using FlowJo v10.
Instrument	Fortessa flow cytometer (BD Biosciences)

Software	FACSDiva (BD Biosciences, version 8.)
Cell population abundance	The purity of sorted populations was dertermined by flow cytometry anaysis of sorted cells and frequencies for the gated population were above 90%.
Gating strategy	Gating strategies were described in Extended Data Fig. 4a.

☒ Tick this box to confirm that a figure exemplifying the gating strategy is provided in the Supplementary Information.
